# Prediction of Deleterious Non-synonymous SNPs of Human STK11 Gene by Combining Algorithms, Molecular Docking, and Molecular Dynamics Simulation

**DOI:** 10.1038/s41598-019-52308-0

**Published:** 2019-11-11

**Authors:** Md. Jahirul Islam, Akib Mahmud Khan, Md. Rimon Parves, Md Nayeem Hossain, Mohammad A. Halim

**Affiliations:** 1 Division of Computer-Aided Drug Design, The Red-Green Research Centre, BICCB, 218 Elephant Road, Dhaka, 1205 Bangladesh; 2 Department of Biochemistry and Biotechnology, University of Science and Technology Chittagong (USTC), Foy’s Lake, Khulshi– 4202, Chittagong, Bangladesh

**Keywords:** Computational models, Molecular modelling

## Abstract

Serine-threonine kinase11 (STK11) is a tumor suppressor gene which plays a key role in regulating cell growth and apoptosis. It is widely known as a multitasking kinase and engaged in cell polarity, cell cycle arrest, chromatin remodeling, energy metabolism, and Wnt signaling. The substitutions of single amino acids in highly conserved regions of the STK11 protein are associated with Peutz–Jeghers syndrome (PJS), which is an autosomal dominant inherited disorder. The abnormal function of the STK11 protein is still not well understood. In this study, we classified disease susceptible single nucleotide polymorphisms (SNPs) in STK11 by using different computational algorithms. We identified the deleterious nsSNPs, constructed mutant protein structures, and evaluated the impact of mutation by employing molecular docking and molecular dynamics analysis. Our results show that W239R and W308C variants are likely to be highly deleterious mutations found in the catalytic kinase domain, which may destabilize structure and disrupt the activation of the STK11 protein as well as reduce its catalytic efficiency. The W239R mutant is likely to have a greater impact on destabilizing the protein structure compared to the W308C mutant. In conclusion, these mutants can help to further realize the large pool of disease susceptibilities linked with catalytic kinase domain activation of STK11 and assist to develop an effective drug for associated diseases.

## Introduction

Single nucleotide polymorphisms (SNPs) are found in every 200–300 base pairs of the human genome and serve as genetic markers^1^. About 0.5 million SNPs are present in the coding region of human genome^2^. Substitution of amino acids in conserved regions of the protein may exert an effect on protein structure, stability, and function. High-risk nonsynonymous SNPs (nsSNPs) are responsible for altering protein function, which cause various diseases in humans^3–5^. Indeed, many studies have reported that ≥50% of the variations linked with hereditary genetic disorders are due to nsSNPs^6–8^. In recent years, nsSNPs in cancer-causing genes have received considerable attention. In various studies, multiple nsSNPs have been identified which influence the probability of infections, and the extension of inflammatory disorders and autoimmune diseases^9–11^. Immunity-related genes are highly polymorphic and many nsSNPs have remained uncharacterized in these genes.

Human STK11 (serine/threonine kinase 11) protein, also known as LKB1 (liver kinase B1), is found at chromosome 19p13.3. STK11 protein is composed of 9 coding exons along with a 433 amino acid long coding sequence and one non-coding exon. The catalytic kinase domain is highly conserved in STK11 protein, which is comprised of residues in 49–309 positions^12^. The STK11 gene acts as a tumor suppressor gene having significant influence in controlling cell growth as well as apoptosis.

A cellular function of STK11 has been regulated through a number of protein-protein interactions. Cell cycle arrest mediated through STK11 is involved in p53-dependent apoptosis pathways as well as in VEGF and Brg1-dependent growth arrest^13–15^. STK11 also has an impact on polarity, metabolism, proliferation in cancer cells through phosphorylation, and activation of AMPK and its related kinases. STK11 forms a heterotrimeric complex *in vivo* with STRADα and MO25α. Both STRADα and MO25α have been involved in relocalization of STK11 from the nucleus to cytoplasm^16^. Cytoplasmic localization is critical for the growth suppressive function of STK11^15^.

Germline mutations in the STK11 gene are responsible for Peutz–Jeghers syndrome (PJS), which is an inherited autosomal dominant disorder and is distinguished by gastrointestinal hamartomatous polyps and mucocutaneous pigmentations. The occurrence of PJS is estimated between 1 in 8300 to 1 in 280000 individuals^17^. The most common malignancy associated with PJS is colorectal cancer, followed by breast, small bowel, gastric, and pancreatic cancers^18,19^. The cumulative lifetime risk of developing cancers in individuals with PJS at ages 20, 30, 40, 50, 60 and 70 years are 2%, 5%, 17%, 31%, 60% and 85%, respectively^17^. Recently, studies have shown that about 57–88% of PJS cases concurrently occur with one or more mutations in STK11 protein, including point mutations and large genomic deletions, duplications, or insertions^20–23^.

Over the past few years, several *in silico* approaches have been developed particularly for screening functional SNPs and detecting the effect of deleterious nsSNPs in the candidate protein. Many tools can also predict the structural changes based on single amino acid substitution in the protein^24,25^. Based on *in silico* algorithms, the functional SNPs in BRAF (B-Raf proto-oncogene), BRCA1(Breast cancer type 1 susceptibility protein)^26^, and ATM (Ataxia-Telangiectasia Mutated) genes^27^ have been classified successfully from a wide range of disease susceptible SNPs based on their functional and structural consequences. Recent computational approaches are focused on cancer studies by involving either in the prediction of the most damaging SNPs from large databases or in population-based data analysis^28–30^.

In this study, detailed investigations have been carried out to postulate 63 nsSNPs in the STK11 protein and to evaluate their deleterious or pathogenic effects on the protein. Employing different prediction algorithms, we classify high-risk nsSNPs and identify their structural and functional impact on the STK11 protein. Moreover, molecular dynamics (MD) and docking calculations are performed to better understand the impact of mutation on protein structure at the secondary and tertiary level.

## Results

**Figure 1:**
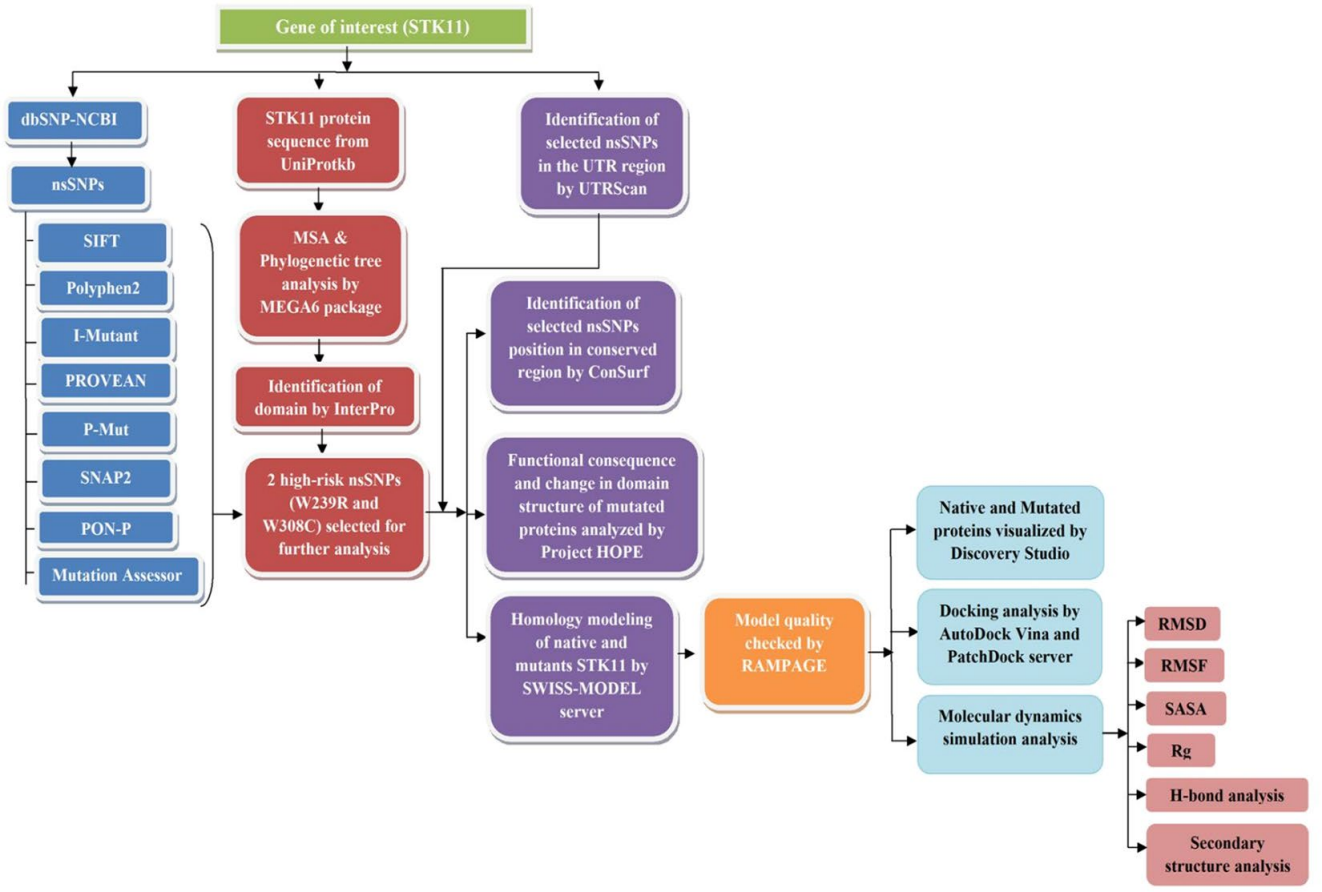
Flowchart for methodology.

The complete workflow, tools, and databases applied to identify the damaging SNPs in human STK11 and their structural/functional consequences due to mutation, are summarized in Fig. 1.

### SNP annotation

**Figure 2:**
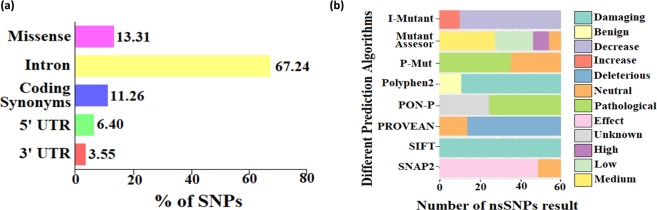
(**a**) Distribution of STK11 missense, coding synonymous, intron, 3′UTR, and 5′UTR SNPs. (**b**) Prediction results of the 63 nsSNPs in the STK11 gene analyzed by the eight computational tools.

The polymorphism information of the STK11 gene was collected from the NCBI dbSNP database, which contains a total of 2283 SNPs for STK11 protein. Out of 2283 SNPs, 1535 are in intron region, 308 are nsSNPs (missense), 257 are coding synonymous, 146 are in 5′ UTR region, and 81 are in 3′ UTR region. It can be noticed from Fig. 2a that most of the SNPs are found in the intron region (67.24%), followed by missense (13.31%), coding synonymous (11.26%), 5′UTR (6.40%), and 3′UTR (3.55%) SNPs, respectively. The interest of the current study is subject to nsSNPs, as they alter the encoded amino acid. Only nsSNPs of STK11 were considered for further analysis.

### Determination of functional SNPs in coding regions

The various computational prediction tools that were used in this study, are illustrated in Fig. 2b to identify significant nsSNPs in STK11. In the SIFT algorithm, 63 nsSNPs are found as deleterious out of 304 missense SNPs that may have a functional effect on the protein. The consequences of SIFT were further evaluated by investigating the impact of nsSNPs in the structure and function of the protein, using PolyPhen2, I-Mutant3.0, and PROVEAN algorithms. In PolyPhen2, 51 nsSNPs are predicted as deleterious. I-Mutant3.0 algorithm predicted 52 nsSNPs that could alter the protein stability due to mutation. PROVEAN predicted 48 nsSNPs as deleterious that could have a functional effect on the protein (Supplementary Table S1). To confirm these results, we further investigated nsSNPs through the *in-silico* prediction pipeline: P-Mut, SNAP2, PON-P, and Mutation Assessor. We noticed 37 nsSNPs to be pathological in P-Mut, 51 nsSNPs as affected in SNAP2, 37 nsSNPs as pathological in PON-P, and 57 nsSNPs to be predicted as disease-associated in Mutation Assessor algorithms (Supplementary Table S2). In this study, a total of eight different computational algorithms were used for the identification of high-risk nsSNPs. By combining the results of all the algorithms, three nsSNPs (W239C, W239R, and W308C) are found to be highly deleterious based on their compared prediction scores. As W239C mutant was reported previously at the 239^th^ position^31^, in this study W239R and W308C mutants were selected for further analysis.

### Identification of domains in STK11

InterPro tool was used to locate domain regions in STK11 and to identify the location of nsSNPs in different domains. This tool provides a functional analysis of proteins by classifying them into families. It also predicts the presence of domains and active sites. It has been reported that three domains: such as the N-terminal domain (1–48), catalytic kinase domain (49–309), and C-terminal domain (310–433) are found in STK11. The two nsSNPs (W239R and W308C) that we have selected are located in the catalytic kinase domain (Supplementary Fig. S1(b)).

### Conservation profile of nsSNPs and evolutionary relationship analysis of STK11 protein

**Figure 3:**
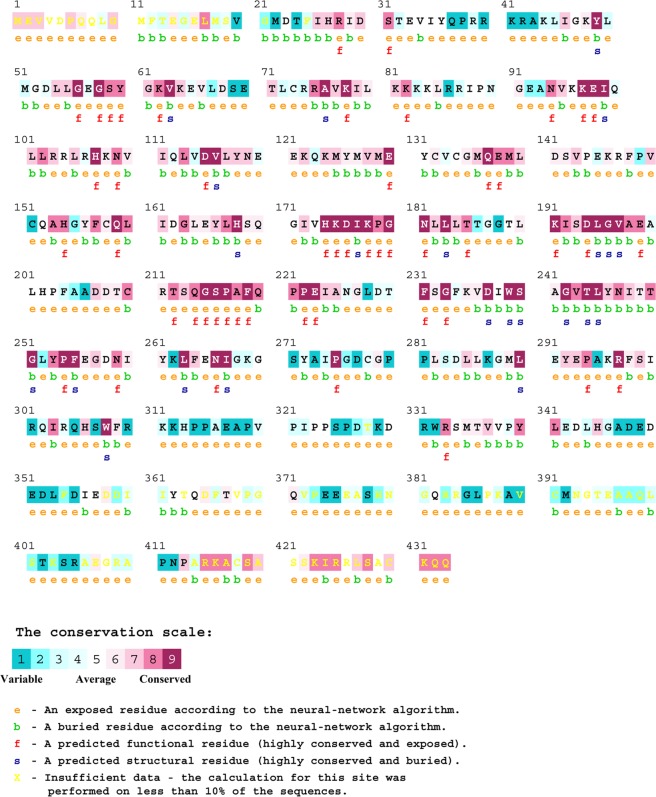
ConSurf analysis of human STK11 residues.

ConSurf web browser was used to measure the intensity of evolutionary conservation at each residue position in STK11 protein. By using the Bayesian method, the ConSurf server recognizes putative functional and structural amino acids and identifies their evolutionary conservation profile^32^. The ConSurf analysis predicted that both mutants, W239 and W308, are buried and conserved residues, *i*.*e*. structural residues (Fig. 3). W239 and W308 residues are involved in the formation of helix-8 and helix-12. Substitution in any of these residues can lead to a decrease in the stability of helix-8 and helix-12^33^. Moreover, this finding is also supported by multiple sequence alignment (MSA) analysis. We performed MSA among the nine different species through use of the MEGA 6 program. It was revealed that conserved regions are homologous in nine species, represented as asterisk signs (“*”) in Fig. S1(b).

The maximal conserved residues are found in the kinase domain, which are essential for stabilizing the secondary structure and activating the STK11 protein. Moreover, phylogenetic analysis was performed using the MEGA 6 package, for understanding the evolutionary relationship among the nine different species. The phylogenetic tree reveals that human (*Homo sapiens*) and chimpanzee (*Pan troglodytes*) are close neighbors (Fig. S1(a)). Dilmeç *et al*. reported that 99% sequence homology is found between human and chimpanzee STK11 proteins^34^. According to the phylogenetic tree analysis, STK11 protein is more conserved in primates, including humans.

### Identification of functional SNPs in the UTR region by UTRscan

Gene expression is inhibited by the SNPs in the 3′ UTR region because of faulty rRNA translation or by affecting RNA half-life^35^. The UTRscan server identifies patterns for regulatory region motifs in the UTR database^36^. This server predicts two UTRsite motifs in the STK11 protein. Three total matches were found for two motifs, as shown in Supplementary Table S3. Here, the W308C mutant was found in upstream open reading frames (uORF) of the 5′UTR region. Mutation at the 308^th^ position in the 5′UTR region of STK11 protein is associated with PJS^37^.

### Structural analysis of native and mutant models

The native structure and two high-risk deleterious variants were modeled by Swiss-Model^38^. Model quality was also validated by generation of a Ramachandran plot using the RAMPAGE server. The Ramachandran plot for both native and mutant models showed 278 residues (94.6%) in the favored region, 11 residues (3.7%) in the allowed region, and only 5 residues (1.7%) in the outer region (Supplementary Figure S2 and Table S4).

### Analysis of structural effects of high-risk nsSNPs in STK11

**Figure 4:**
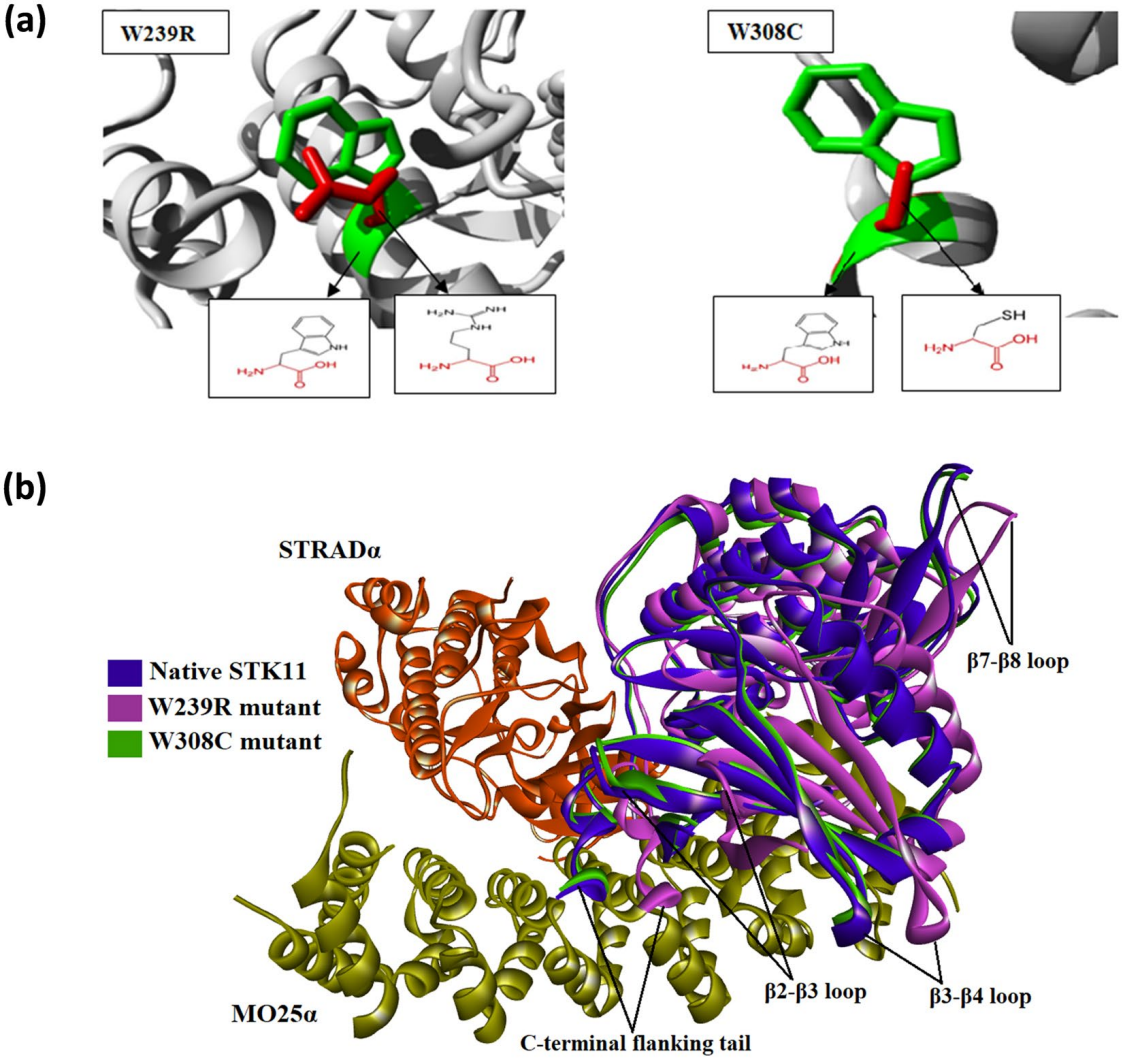
(**a**) Superimposed structures of STK11 native (green color) and mutant (red color) models to visualize the stereochemical conformation of wild type and mutant residues at 239 and 308 positions (**b**) Superimposed image of native and mutant STK11 proteins docked against STRADα-MO25α protein complex.

The impact of amino acid substitutions on the domain structure of STK11 were investigated using the Project Hope server. The W239R variant results in an arginine residue in place of tryptophan at the 239^th^ position located in the kinase domain. This domain is important for protein binding and replacement of the buried neutral tryptophan residue with positively charged arginine that may cause an empty space in the core structure of the domain and can lead to protein folding problems. A graphical view of the variant is shown in Fig. 4a. A similar destabilizing condition is formed by the W308C mutant. Replacing a buried structural tryptophan with a smaller cysteine residue can create an empty space in the core structure of the kinase domain that may affect the signal transduction between the domains, as shown in Fig. 4a. Mutation in the kinase domain is hypothesized to be responsible for disrupting the binding activity of STK11 because both N and C-terminal lobes of the STK11 kinase domain interact with the C-terminal lobe of STRADα^33^.

### Docking Analysis

**Figure 5:**
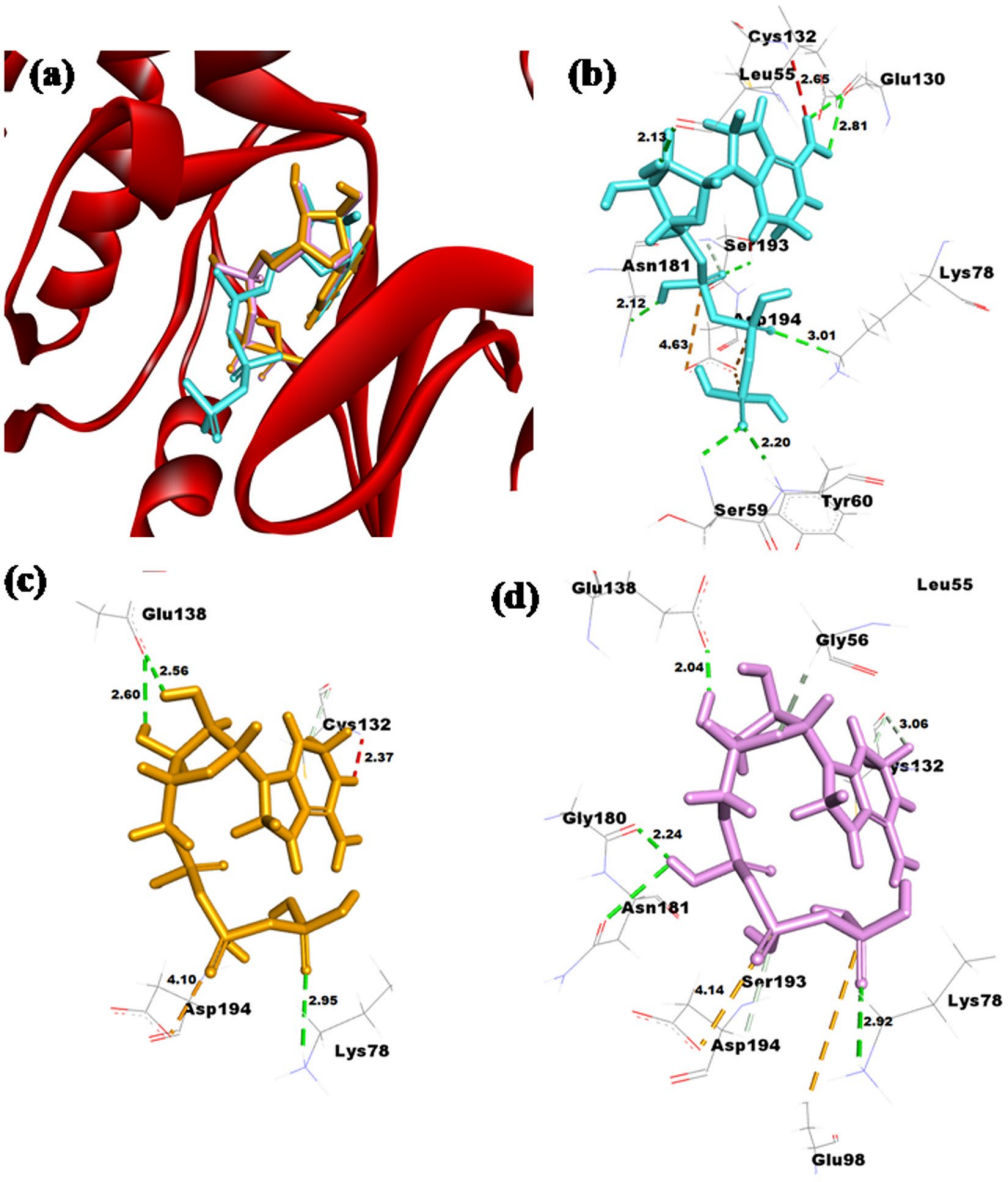
(**a**) Superimposed image of ATP docked against native and mutant STK11 protein and interaction of ATP with (**b**) native, (**c**) W239R mutant, and (**d**) W308C mutant STK11 protein residues.

Protein-protein docking analysis demonstrated that the mutant STK11 structures bind to the STRADα-MO25α complex in a slightly deviated orientation compared to the native STK11 structure. The W239R mutant shows higher deviation compared with the W308C mutant Fig. 4b. The C-terminal flanking tail, β2-β3 loop, β3-β4 loop, and β7-β8 loop of the W239R mutant showed significant deviation in orientation. Molecular docking of ATP with native and mutant modeled structures also showed a difference in binding affinity. The binding affinity of ATP with native STK11 is −7.7 kcal/mol, while for mutant W239R and W308C are −7.1 kcal/mol and −7.2 kcal/mol respectively. ATP binds at the same binding pocket when compared in native and mutant proteins; however, from the analysis of the binding pose of ATP, a significant deviation in terminal phosphate of ATP is observed between native and mutant protein complexes Fig. 5. Interaction analysis of ATP with native and mutant protein showed a reduction in the number of hydrogen bonds and attractive electrostatic charge interactions of ATP with residues in mutant proteins (Table 1). Many residues such as L55, S59, Y60, E130, and S193 have interactions with ATP in native STK11 but were absent in mutant proteins.

**Table 1:** Interactions of ATP with native and mutant STK11 proteins. (AC = Attactive electrostatic Charge interaction; H = Hydrogen bond; C = Carbon Hydrogen bond; UDD = Unfavorable Donor-Donor clash).

Interacting Residue	Distance (Å)	Bond Type	Interacting Residue	Distance (Å)	Bond Type	Interacting Residue	Distance (Å)	Bond Type
Native STK11			Mutant W239R			Mutant W308C		
Asp194	3.806	AC	Asp194	4.100	AC	Glu98	5.583	AC
Asp194	4.629	AC	Lys78	2.949	H	Asp194	4.137	AC
Asp194	4.039	AC	Glu138	2.600	H	Lys78	2.918	H
Ser59	2.431	H	Glu138	2.556	H	Glu138	2.035	H
Tyr60	2.196	H	Cys132	2.705	C	Gly180	2.242	H
Lys78	3.006	H	Cys132	2.372	UDD	Asn181	2.907	H
Ser193	2.379	H				Gly56	2.374	C
Glu130	2.573	H				Asp194	3.001	C
Glu130	2.812	H				Cys132	2.660	C
Leu55	2.131	H				Cys132	3.062	C
Asn181	2.124	H						
Ser193	2.848	C						
Cys132	2.646	UDD						

### Molecular dynamics simulation of native and mutant STK11 proteins

**Figure 6:**
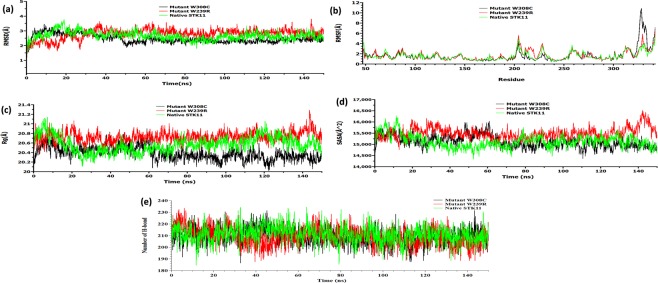
Analysis of RMSD, RMSF, Rg, and SASA of native and mutant STK11 structures at 150 ns. (**a**) RMSD values of Cα atoms of native and mutant structures. (**b**) RMSF values of the carbon alpha over the entire simulation. The ordinate is RMSF (Å), and the abscissa is residue. (**c**) Rg of the protein backbone over the entire simulation. The ordinate is Rg (Å), and the abscissa is time (ns). (**d**) The ordinate is SASA (Å^2^), and the abscissa is time (ns). (**e**) Total number of H-bond count throughout the simulation of native and mutant structures. The symbol coding scheme is as follows: native (green colour), mutant W239R (red colour), and W308C (black colour).

150 ns MD simulations were performed to study the deviation of native and mutant proteins in physiological environments. Native STK11 and mutant W308C have similar root mean squared deviation (RMSD) values with significant deviation observed for the W239R mutant, as shown in Fig. 6a. Mutant W239R presented an increasing trend in RMSD value throughout 36.9 ns (RMSD ~3.611 Å) and 140 ns (RMSD ~3.76 Å) of MD simulation. Average RMSD values of native, W239R, and W308C mutants are 2.67 Å, 2.80 Å and 2.47 Å, respectively. In addition, RMSF (root-mean square fluctuation) value analysis also shows residues in C terminal region have a significant difference in fluctuation between native and mutant structures after 150 ns MD simulation (Fig. 6b) with RMSF values reaching as much as 7 Å for mutant W239R. From the RMSF plot, it can be observed that residues in 204–228 positions constitute a comparatively flexible region than other residues in the W239R mutant. On the other hand, the highest residual fluctuation can be observed at positions 327 (10.21 Å) and 328 (10.77 Å) in mutant W308C when compared to the native structure (Fig. 6b). Furthermore, it can be noticed that in the mutant W308C, fluctuation of residues (322–334) in C-terminal flanking tail is greater, with values ranging from 2.13 to 10.71 Å when compared to that in the native type (from 1.25–3.80 Å). Deviation in the position and conformation of the bound ATP molecule can be observed when compared between the native and mutant protein complexes (Fig. 5) with a significant deviation observed between the native and mutant W308C.

From Rg (radius of gyration) analysis of native and mutant structures (Fig. 6c), it can be observed that the W239R mutant revealed a higher average Rg value (20.73 Å) over the simulation time scale when compared to those of native STK11 (20.56 Å) and mutant W308C (20.38 Å). As a result, the flexibility of mutant W239R may be increased. On the other hand, mutant W308C seemed to deviate its Rg value after 60 ns, which could be the reason for its partial protein folding.

Solvent accessible surface area (SASA) analysis indicated that mutant W239R has a higher average SASA value (15498.11 Å2) than mutant W308C (15121.88 Å2) and native STK11 (15129.68 Å^2^), as shown in Fig. 6d. Since a higher SASA value denotes protein expansion, it can be suggested that mutant W308C and native STK11 are more stable than the mutant W239R. The reason for a greater change observed in the SASA value of W239R compared to native STK11 could be the effect of amino acid substitution by altering the size of the protein surface and other characteristics^39,40^.

The total number of H-bonds within the proteins were also calculated during the MD simulation as depicted in Fig. 6e. From the analysis, it can be noted that the native structure forms a greater number of H-bonds with an average of ∼212, while W239R and W308C mutants exhibit a fewer number of H-bonds with an average of for each mutant of ∼209. Since the number of H-bonds was less in the mutant structures, protein stability may be effected.

**Figure 7:**
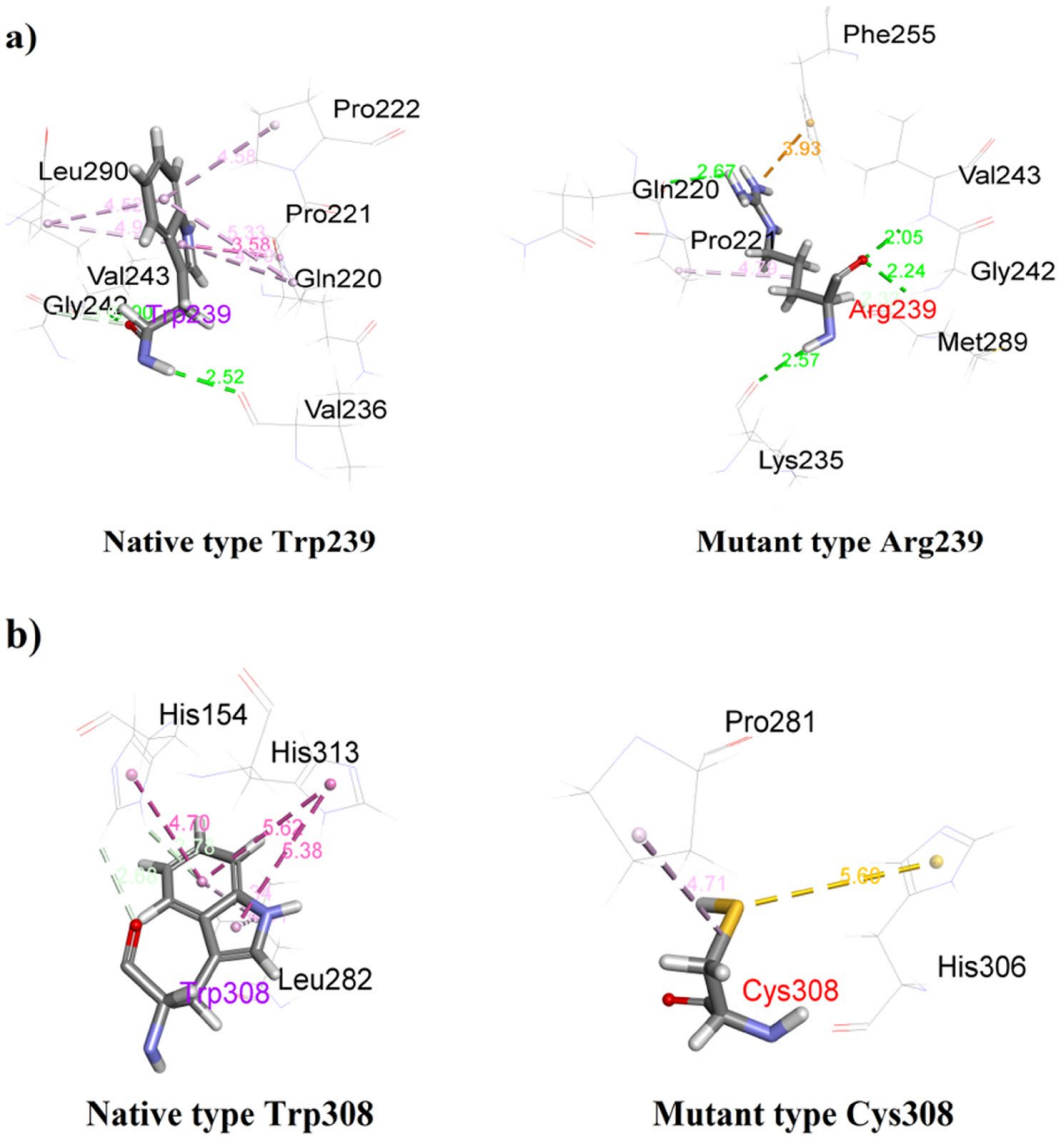
Non-bonding interactions of native type STK11 and mutant STK11 proteins at 239 and 308 positions with other residues.

To further understand the interaction of native and substituted residues with surrounding residues, we analyzed non-bonding interactions after MD simulation. Mutant W239R showed a number of interactions with the surrounding residues which are significantly decreased due to the substitution of Trp with Arg at the 239^th^ position, as shown in Table 2 and Fig. 7a. This decrease is largely due to the failure to form any hydrophobic interactions such as pi-alkyl interaction by R239 in the mutant structure. H-bond interaction with V236 is absent in the mutant type but forms new H-bond interactions with Q220 and K235. R239 also forms a pi-cation interaction with F255, whereas W239 in the native structure does not form any interaction with this residue. On the other hand, a number of hydrophobic interactions such as pi-pi stacked, pi-pi T-shaped, and pi-alkyl are significantly decreased due to the substitution of Trp with Cys at position 308 (Table 2 and Fig. 7b). Moreover, a pi-sulfur and an alkyl interaction with H306 and P28, respectively are present in the W308C mutant, but absent in native type.

**Table 2:** Non-bonding interaction analysis.

Interacting Residue	Distance (Å)	Bond Type	Interacting Residue	Distance (Å)	Bond Type
Native Trp239			Mutant Arg239		
VAL236	2.52057	H	LYS235	2.5719	H
**VAL243**	**1**.**89825**	H	GLN220	2.66571	H
GLY242	3.09067	CH	GLY242	2.23827	H
GLN220:PRO221	3.57802	Amide-Pi Stacked	**VAL243**	2.05125	H
PRO221	4.69626	Pi-Alkyl	MET289	2.31749	CH
LEU290	4.94724	Pi-Alkyl	PHE255	3.93234	Pi-Cation
PRO221	5.33427	Pi-Alkyl	PRO221	4.78627	Alkyl
PRO222	4.58115	Pi-Alkyl			
LEU290	4.52331	Pi-Alkyl			
**Native Trp308**			**Mutant Cys308**		
HIS154	2.68372	CH	HIS306	5.59859	Pi-Sulfur
HIS154	2.78251	Pi-Donor Hydrogen Bond	PRO281	4.70969	Alkyl
HIS313	5.38136	Pi-Pi Stacked			
HIS313	5.61516	Pi-Pi Stacked			
HIS154	4.69812	Pi-Pi T-shaped			
LEU282	4.80756	Pi-Alkyl			
LEU282	4.34223	Pi-Alkyl

**Figure 8:**
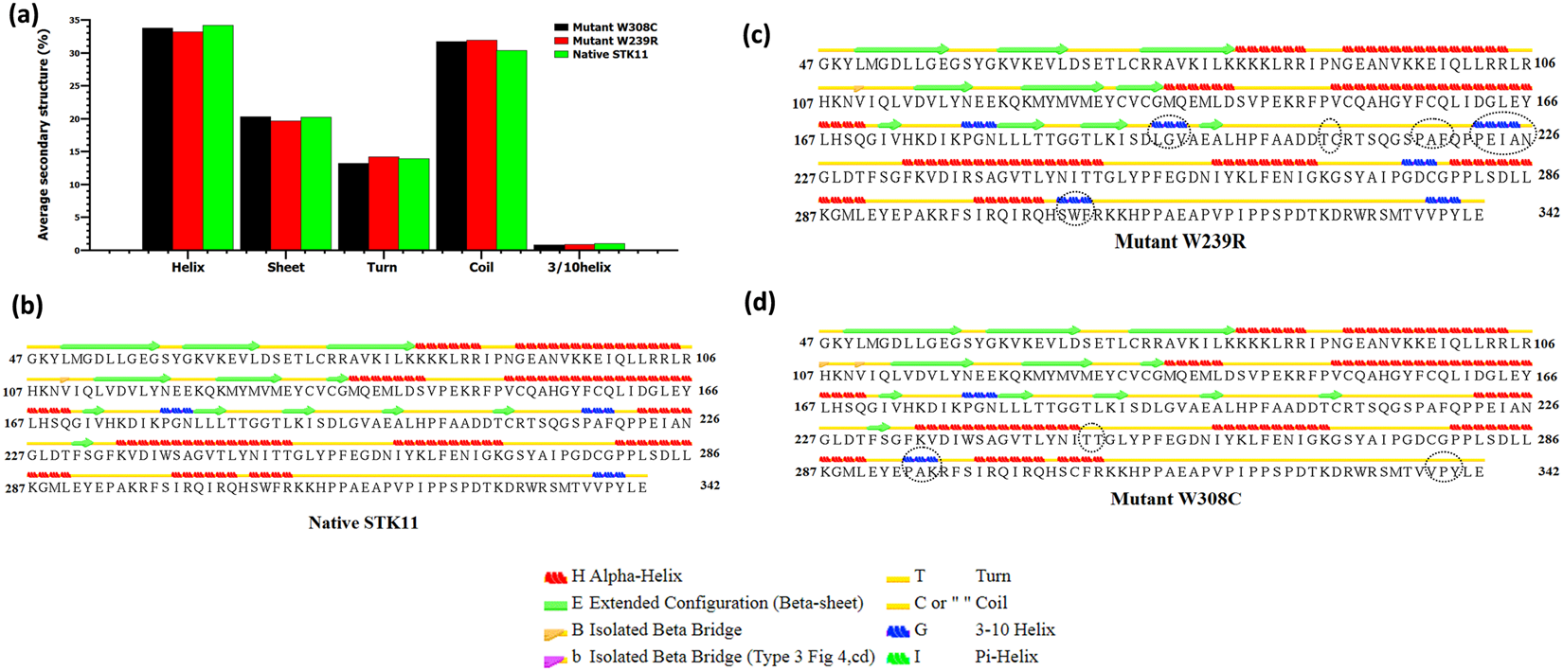
Average Secondary structure of native and mutants (**a**). Secondary structural elements of native STK11 (**b**) and mutant STK11 (**c**,**d**) proteins are analysed.

Additional information on the flexibility of STK11 mutation was acquired by the analysis of secondary structures of native, W239R and W308C variants during 150 ns MD simulation as presented in Fig. S3. The average percentage values of secondary structures of native and mutant proteins are close in approximation. In Fig. 8, the average alpha helix values of native, W239R, and W308C mutants are 34.23%, 33.22%, and 33.78%, respectively. Although, alpha helix showed a decreasing trend in W239R mutant between 35–85 ns, after that it was stable (Fig. S3a). The average sheet and turn percentage values were found to be similar for native (20.29% and 13.97%) and W308C (20.34% and 13.27%), but in W239R, slightly different values were found (19.67% and 14.24%) (Fig. S3b). In Fig. S3c, the β turn exhibited an increasing trend in the W239C mutant between 45–87 ns when compared to the native protein and W308C mutant. Average coil percentage values of native, W239R and W308C structures were determined to be 30.40%, 31.97%, and 31.76%, respectively (Fig. S3d). Secondary structure analysis also showed that residues in the coil region have a significant difference in fluctuation between the native and mutant structures after 90 ns MD simulation. The average 3_10_-helix percentage values for native STK11, W239R, and W308C are 1.08%, 0.88%, and 0.81%, respectively (Fig. S3e).

**Figure 9:**
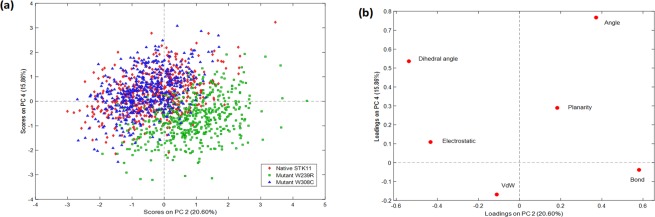
(**a**) The scores plot presented three data clusters in different color, where each dot represented one time point. The clustering is attributable to the three different proteins: native STK11(red), mutant W239R(green), and mutant W308C(blue) proteins. (**b**) Loadings plot from Principal Components Analysis of the energy and structural data.

Energy and structural information of three protein structures (native STK11, mutant W239R, and mutant W308C) were analyzed using principal component analysis (PCA) to understand the structural quality of proteins during MD simulation. The energy and structural information are the composition of the following variables; bond energies, bond angle energies, dihedral angle energies, planarity energies, Van der Waals energies, and electrostatic energies. The exploratory PCA analysis of these variables revealed the similarity and dissimilarity between native and mutant proteins in the scores plot (Fig. 9a). The scores plot showed that the distance between native STK11 and mutant W239R was further whereas native STK11 and mutant W308C was overlapped. Dihedral angle energies and bond energies loaded significantly into the second PC resulting in the dissimilarity of mutant W239R with respect to native STK11 and mutant W308C (Fig. 9b).

We analyzed position level variations in secondary structure elements of native and mutants using the STRIDE program. In the native complex, helix regions (residues 222–226 and 307–310) are disrupted and formation of 3_10_-helix was observed in mutant W239R (Fig. 8). Furthermore, the most prominent alteration was observed in residues 195–197 and 276–278 positions due to an acquisition of 3_10_-helix conformation in mutant W239R. Moreover, two strands (residues 209–210 and 231–232) are present in native STK11, whereas these strands have converted to coils in mutant W239R. On the other hand, W308C mutant showed variation at positions 217–219 and 338–340, which transformed from 3_10_-helixes to turns, and also showed variation by converting to a 3_10_-helix from a turn at position 294–296 (Fig. 8). Furthermore, an alpha helix (residues 235–250) is found slightly reduced in the W308C mutant structure.

## Discussion

All *in silico* prediction algorithms disclose two nsSNPs, W239R and W308C, that are highly deleterious based on their compared prediction scores. Typically, conserved residues are involved to control the biological system in proteins like stability and/or folding^41^. Functional amino acids are located at enzymatic sites and show substantial protein-protein interaction. These residues are more highly conserved compared to other residues in the protein^42^. For assessing the deleterious impact of two nsSNPs (W239R and W308C) in the STK11 protein, we estimated the evolutionary conservation profile of all amino acids position in STK11 protein via ConSurf web server. This server predicted that tryptophans at the 239^th^ and 308^th^ positions are buried structural residues with a conservation score of 9. For evaluating ConSurf result, we executed the multiple sequence alignment (MSA) among the nine different species through the MEGA 6 program, which revealed that the conserved regions were homologous in nine species. Maximum conserved residues are found in the kinase domain, which is essential for stabilizing the secondary structure and in activating the STK11 protein. Moreover, the phylogenetic tree reveals that human and chimpanzee are close neighbors. One study reported that 99% sequence homology is found between the human and chimpanzee STK11 proteins^34^. This result indicated that the catalytic kinase domain is evolutionarily conserved in STK11 protein.

In the catalytical kinase domain, the W239R mutant leads to substitution of tryptophan (a nonpolar aromatic amino acid) by arginine (a basic amino acid). Although the mutated residue is smaller in size than the wild-type residue, it is likely to disturb the interaction between other domains that are important for the protein activity as predicted by Project HOPE. Mass and charge difference in the protein affect spatiotemporal dynamics of protein-protein interactions^43,44^. The mutation introduces a charge which may lead to repulsion between the mutant residues and neighboring residues^44^. Hence, this variant altered the binding interaction with surrounding residues, thereby disturbing normal biological processes. The mutant W308C involves the substitution of tryptophan to cysteine, where tryptophan is larger in size than cysteine. Moreover, a buried structural tryptophan residue is more hydrophobic than the cysteine residue, which probably will not fit at the 308^th^ position. As a result, this variant will cause a loss of hydrophobic interactions in the core of the protein, as predicted by ProjectHOPE. Structural mutations affect buried residues in the protein core, causing changes in amino acid size and charge, hydrogen bonds, salt bridges, and S–S bridges. These changes cause loss of thermodynamic stability as well as aberrant folding and aggregation of the proteins^45^.

To further evaluate our hypothesis as to whether W239R and W308C mutants have a deleterious effect on STK11 protein, we performed molecular docking and MD simulation analysis. From docking analysis of STK11 with ATP, it is well revealed that both mutants perturbed the binding pocket quite significantly. The most prominent change was noticed in W239R where a significant loss of H-bond interactions within the binding pocket residues can be observed when compared to that in the native protein. In the STK11-ATP complex, ATP binds to a cleft between N- and C-lobes of the protein kinases through formation of H-bonds with the glycine-rich loop (G58-K62), β3 strand (K78), the hinge regions (E130-C132), αD-helix (E130-M136), the catalytic loop (H174-N181), and the activation loop (S193-A198)^46,47^. ATP is strongly bound to the binding cleft of STK11, so these mutants disrupt the favorable contacts which are essential for the functional activity of STK11. Moreover, the deviation observed in the bound ATP molecule can lead to a reduction in catalytic efficiency of STK11.

**Figure 10:**
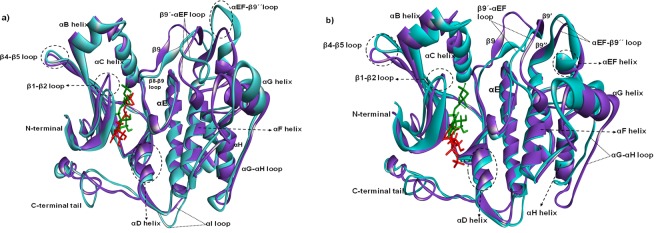
Superimposed structures of (**a**) native STK11 and mutant W239R and (**b**) native STK11 and mutant W308C proteins after 150 ns MD simulation. (Light violet: native STK11; Cyan: Mutants) The symbol coding scheme is as follows: native (light violet colour) interaction with ligand (green colour), mutants (cyan colour) interaction with ligand (red colour).

Proteins are dynamic entities in an aqueous environment. We performed 150 ns MD simulations to observe the effect of mutation on the structural dynamics of the STK11 protein. RMSD values remained relatively constant for both native STK11 and W308C mutant, indicating that the W308C mutant is likely to form a stable structure in physiological conditions; however, consistently higher RMSD values throughout the MD simulation for the W239R mutant indicate that this mutation is likely to make the protein structure less stable. Furthermore, higher fluctuation and a loss of stability were observed for the W239R mutant in RMSF, Rg, SASA, and H-bond analysis. This feature was also confirmed by the principal component analysis (Fig. 9a). The deviation observed after MD simulation in the F204-L228 residue loop region supports our earlier hypothesis that the positively charged R239 residue is likely to disrupt the interactions with surrounding residues. Non-bonding interaction analysis revealed that the residues Q220, P221, and P222 are directly in contact with native W239. Furthermore, L201-A206 (β9-β9′ loop) from the STK11 activation loop have strong interactions with a hydrophobic pocket on the concave surface of MO25α^33,46^. The disruption in the activation loop regions (D194-E223) may cause inhibition in activation of the STK11-MO25α complex. On the other hand, the W308C mutant showed a stable structure like the native type, but higher residual fluctuation can be observed in the residues (I322-S334) of the C-terminal flanking tail. The W308C mutant lost five polar contacts with H154 and H313 and one hydrophobic contact with L282 (Table 2). Polar contacts are important, as they are involved in the formation of H-bonds and these H-bonds and hydrophobic interactions help in maintaining the stability of the protein^48,49^. A Cys residue inside the polypeptide chain has the possibility to form a disulfide bond with another Cys residue and can alter the tertiary structure of the protein. Mehenni *et al*. have reported that C308 is likely to form a disulfide bond with C158. During the folding of STK11, this interaction may result in divergence of the tertiary structure with slight or no kinase activity^37^. We executed secondary structure analysis for a definitive understanding of the disruption in the native and mutant secondary structures over time. As shown in Fig. 8 and **S3**, a significant difference is observed in alpha helix, beta sheet, beta turn, and coil regions for both mutants when compared to the native structure. We analyzed the position level variations in secondary structure elements of native and mutants through use of the STRIDE program. The most significant difference is observed in mutant W239R, from the amino acid residue positions of L195-V197 (from β8-β9 loop), P217-F219 (from β9΄-αEF loop), P221-I224 (from αEF-helix), G276-C278 (from αG-αH loop), and S307-F309 (from αI-helix), as shown in Fig. 8c and Fig. 10a. Furthermore, T209-C210 (β9΄strand) is totally disrupted in the mutant W239R structure. In the native structure, the αEF-helix is comprised by P222-N226 residues and the αI-helix is comprised by I300-R310 residues, whereas P221-I224 (from αEF-helix) and S307-F309 (from αI-helix) are converted to 3_10_-helix in mutant W239R structure, as shown in Fig. 8d. The conformational changes support our previous results obtained from RMSF analysis that major changes occurred at residues 204–228 in the W239R mutant (Fig. 6b). The co-activator protein, MO25α express strong interactions with E165, S169, Q170, G171 (from αE-helix), L201-A206 (β9-β9′ loop), R301, S307, and R310-K312 (from αI-helix) of STK11^33,46^. Furthermore, H174-D176 motif, β7, and β8 sheets are essential for forming the catalytic region. The activation loop segment is formed by D194-G196 motif, β9, β9′, and β9′′ and ends at P221-E223 motif, in which H174 from H174-D176 motif and D199 from D194-G196 motif are coordinated to Mg^2+^ along with PO_4_^−^ ions of ATP^46^. Consequently, the W239R mutant changed the conformation of the C-lobe in the kinase domain (Fig. 10a) and could disrupt the STK11-MO25α interaction, which might have an impact on complex assembly as well as could suppress the STK11 activation^33^.

In the W308C mutant structure, the most prominent alteration was observed from the amino acid residue positions of P294-A295 (from αH-αI loop) and V338-Y340 (from the C-terminal tail) acquiring a 3_10_-helix conformation (Figs 8d and 10b). Interestingly, these 3_10_-helix conformations are only observed in the W308C mutant structure, but are not present in the native type. Furthermore, C134-G135 (from β5-αD loop) is converted to a β-strand in the mutant structure. The proportion of αC-helix (94–104), αF-helix (234–250), and αI-helix (300–310) are slightly reduced in the W308C mutant structure when compared to those in the native structure (Fig. 10b). Binding of both STRADα and MO25α reorients the αC-helix of STK11 in such a way that a salt bridge is formed between K78 of the VAIK motif and E98 of the αC-helix^33^. The regulatory spine is comprised of four hydrophobic residues such as L113, L102, H174, and L195, which are anchored to D237 of the αF-helix^46^. R301, S307, and R310-K312 (from the αI-helix) of STK11 interact with MO25α^33,46^. Boudeau *et al*. reported that mutations located at positions L67, F157, K175-L182, W239-G242, and R297-W308 residues of STK11 abolished binding to STRADα and MO25α^16^. Scott *et al*. were first to identify the W308C mutant in a 42-year old patient with PJS^31^.

From PCA analysis, mutant W239R showed a significant difference in flexibility than native and W308C mutant. This deviation might be due to disruption of secondary structure elements, which in turn affect the protein folding, thereby decreasing the stability of protein. Therefore, we suggest that the W239R mutant might have a great impact on protein function. This hypothesis is in good concordance with the results obtained by Rungsung *et al*.^50^.

## Conclusion

This study reports two nsSNPs (W239R and W308C) that were found to be deleterious and have a mutational impact on structure and function of the STK11 protein. These mutations may lead to disruption of the original conformation of the native protein. The mutant W239R structure displays a significant difference in binding with the STRADα-MO25α complex, which could lead to disruption in activation and reduction in catalytic efficiency of the STK11 protein. Our molecular dynamics approach presented a change of deviation in important regions of the mutant structures when compared to the native protein structure. These deviations can interrupt the confirmation of the secondary structure, and thereby, the stability of protein may be disrupted. We also noticed that the ATP binding capability of the mutant proteins was less than that of the native protein. Although the W308C mutant has been previously associated with Peutz-Jeghers Syndrome (PJS) according to the literature, no one has predicted the W239R mutant to be linked with any diseases. Therefore, it is likely that predisposition of the unreported nsSNP can induce disease by altering protein activation or efficiency. The findings of this study will help in future genome association studies to distinguish deleterious SNPs associated with different individual patients with PJS. Hence, comprehensive clinical-trial-based studies are required on a large population to characterize this data on SNPs and also experimental mutational studies are required for the validation of the findings.

## Materials and Methods

### Collection of SNPs dataset

The SNP data for the human STK11 gene was collected from various web-based data sources such as: OMIM (Online Mendelian Inheritance in Man)^51^, SNPs information from NCBI dbSNP^52^, and the protein sequence was retrieved from UniProt database (UniProtKB ID Q15831)^53^.

### Assessment of functional consequences of missense mutations

Functional consequences of nsSNPs in the coding region were predicted by using SIFT, PolyPhen 2, I-Mutant 3.0, PROVEAN, P-Mut, SNAP2, PON-P, and Mutation Assessor algorithms. SIFT uses the homology sequence of the proteins and alignment of natural nsSNPs with orthologous and paralogous protein sequences to predict nsSNPs as deleterious. A SIFT score less than 0.05 indicates deleterious impact of nsSNPs on protein function^54^. Another algorithm, PolyPhen2, utilizes the protein sequence and substitution of amino acids in protein sequence to predict the structural and functional effect on the protein. If amino acid are changed or a mutation is found in protein sequence, it classifies SNPs as possibly damaging (probabilistic score >0.15), probably damaging (probabilistic score >0.85), and benign (remaining). Furthermore, PolyPhen2 calculates the position-specific independent count (PSIC) score for each variant in protein. The difference of PSIC score between variants indicates that the functional influence of mutants on protein function directly^55^. The Protein Variation Effect Analyzer (PROVEAN) algorithm was used to predict the damaging effect of nsSNPS in the STK11 protein sequences. This tool utilizes delta alignment scores on the basis of the variant version and reference of the protein sequence regarding the homologous sequences. An equal score or below the threshold of −2.5 indicates deleterious nsSNP alignment^56^. The change in stability caused by mutation was predicted by I-Mutant 3.0. It is a support vector machine (SVM) based tool server. I-Mutant 3.0 prediction is classified into three categories, such as neutral mutation (−0.5 ≤ DDG ≥ 0.5 kcal/mol), large decrease (<−0.5 kcal/mol), and large increase (>0.5 kcal/mol). I-Mutant predicts the Gibbs free energy change (ΔG) dependent on the difference of mutated protein and native type protein. PMUT allows the fast and accurate prediction (~80% success rate in humans) of the pathological character of single amino acid point mutations based on the use of neural networks. Inputing a FASTA sequence in the PMut server provided results which showed the difference among neutral variations and disease-related protein sequence. A prediction score more than 0.5 indicates nsSNPs having a pathological impact on protein function^57^. SNAP2 is a neural network based classifier. It was used to predict the functional impact of single amino acid substitutions in the STK11 protein. This server accepts a FASTA sequence and provides a prediction score (ranges from −100 strong neutral prediction to +100 strong effect prediction) that reflects the likelihood of a specific mutation to alter the native protein function^58^. Another algorithm, PON-P2, predicts the pathogenicity (harmfulness) of amino acid substitutions. It is a machine learning-based tool and utilizes amino acid properties, GO annotations, evolutionarily conserved sequence and functional annotations. PON-P2 distributes variants into 3 groups: pathogenic, neutral, or unknown classes^59^. The Mutation Assessor algorithm predicts the functional impact of amino acid substitutions. The functional impact is evaluated based on evolutionary conservation of the affected amino acid in protein homologs^60^.

### Identification of mutant nsSNPs position in different domains

The InterPro (http://www.ebi.ac.uk/interpro/) tool was used for identification of different conserved domains in the STK11 protein and also mapping of nsSNPs positions in different domains^61^. Protein sequence in FASTA format or protein ID was inserted as a query to predict domains and motifs.

### Identification of functional SNPs in conserved regions and Phylogenetic analysis of STK11

Amino acid substitutions in the evolutionarily conserved region were predicted by the ConSurf server^32^. According to the Bayesian method, conservation scores were classified into 3 categories: 1–4 score is variable, 5–6 score indicates intermediate, and 7–9 score is conserved^62,63^. A protein structure or protein FASTA sequence of STK11 was input and conserved patterns were predicted in order to find a conservation score and colouring scheme. Structural and functional amino acids were also predicted. High-risk nsSNPs residing in the highly conserved region were selected for further analysis.

To execute phylogenetic analysis for human STK11 protein sequence (UniProtKB ID STK11_HUMAN) and eight different species protein sequences such as *Macaca mulatta* (H9ETP1_MACMU), *Pan troglodytes* (H2QET9_PANTR), *Gallus gallus* (STK11_CHICK), *Rattus norvegicus* (STK11_RAT), *Mus musculus* (STK11_MOUSE), *Xenopus tropicalis* (F6PM85_XENTR), *Bos taurus* (E1BCU9_BOVIN), and *Ovis aries* (W5PNM2_SHEEP) were retrieved from the UniProtKB and were subjected to evolutionary conservation. Then, multiple sequence alignment (MSA) was executed by the ClustalW tool and the phylogenetic tree was created using 1,000 bootstraps in the MEGA6 package^64,65^.

### Identification of high-risk nsSNPs in the UTR regions

The 5′ and 3′UTRs have significant roles in the post-transcriptional regulation of gene expression, message stability, translational efficiency, and subcellular localization^66^. UTRScan was used to predict high-risk nsSNPs in UTRs^67^. Its input format requires submission of a protein′s FASTA format. Output was in the form of signal name and its position in the transcript.

### Modeling of native and nsSNPs structure

The mutant SNPs can notably alter the stability of proteins. Consequently, 3D structures of native and mutant proteins were constructed to investigate the structural stability and deviations difference within native and mutant proteins. We generated the 3D structure of native and mutant proteins using the SWISS-MODEL server^38^. Only the protein kinase domain of the proteins was modeled which is comprised of residues 47–342. Structural validation was carried out by the RAMPAGE server^68^ and the 3D structures were visualized by Discovery Studio 4.1^69^.

### Prediction of structural effects upon mutation

Project HOPE web server (http://www.cmbi.ru.nl/hope/home) was used for predicting the structural impact of amino acids substitutions on the STK11 protein. To evaluate the structural features of mutations on the native protein, Project HOPE utilized the tertiary structure of the proteins that are available in the Distributed Annotation System (DAS) servers and Uniprot database. If necessary, the Project HOPE web server can build homology models separately. This server also provides significant information about the structural changes between mutant and native residues^70^.

### Molecular docking analysis of *STK11*

Native and mutant modeled STK11 structures were docked against the STRADα-MO25α complex. To obtain the STRADα-MO25α complex all the chains and hetero atoms in the PDB file of PDB ID: 2WTK were removed except chain A and B which corresponds to MO25α and STRADα proteins respectively. This complex was loaded as a receptor molecule in the PatchDock server^71,72^ and modeled structures were loaded as ligand molecules. Clustering RMSD was set at 4 Å, complex type as default, then protein-protein docking was performed. The solution with the highest score was chosen for further analysis. Docked complexes of STK11-MO25α-STRADα were aligned and visualized in Discover Studio 4.1^69^. The binding affinity of ATP with the native and mutant STK11 structures was also evaluated. Quantum chemical calculations were conducted using the Gaussian 09 program package^73^. The ATP structure was retrieved from PubChem database (PubChem CID: 5957) and was optimized in the ground state by DFT (density functional theory) using the B3LYP 6–31 G(d) basis set. Docking analysis of native and mutant proteins with ATP was performed by AutoDock Vina^74^. In AutoDock Vina, the parameters were set at default and the grid box was set such that it covered the full structure of the proteins. Torsional rotation of all rotatable bonds was allowed for ligands and molecular docking was performed. After that, the binding complex of target proteins and ligand were obtained by PyMoL^75^ and visualized in Discovery Studio 4.1^69^.

### Molecular Dynamics Simulation

MD simulations were performed using the YASARA dynamics program^76^ to reveal changes at the atomic level in different time scales for native and mutant STK11-ATP complexes. Before starting the simulation, the structures of native and mutant proteins were cleaned and also the H-bond network was optimized^77^. Then, a cubic cell was formed by extending 8 Å on each side of the protein and a periodic boundary condition was maintained. The AMBER14 force field was applied for simulations^78^. The system was implemented by adding water molecules and NaCl salt at 0.9% concentration to replicate the physiological ion concentration. For short-range Coulomb and van der Waals interaction, the cut-off radius was set to 8 Å. The long-range electrostatic interactions were measured by the PME (particle-mesh Ewald) method^79^. MD simulation of each system was run for 150 ns at 310 K with a time step interval of 2.5 fs. The trajectory files were evaluated to get RMSD, RMSF, Rg, SASA, H-bond, and secondary structure analysis.

### Principal component analysis (PCA)

The PCA method projects multivariate energy factors into low-dimensional space, which highlights the variabilities present in the collected MD trajectory data although they do not specifically aim to identify them.^80,81^ Any potential energy variabilities due to hidden data structures such as groups of samples, local fluctuations in data densities, and unique samples can be visualized by this technique. PCA decomposes the multivariate response arranged in an X matrix into a product of two new matrices as indicated in the following equation:

$$X={T}_{k}{P}_{k}^{T}+E$$

where *T*_*k*_ is the matrix of scores which represents how sample relate to each other, ${P}_{k}\,\,$is the matrix of loadings which contains information about how variables relate to each other, *k* is the number of factors included in the model and *E* is the matrix of residuals, which contains the information not retained by the model. Mutant proteins may have different energy profile compared to the native protein which should be discernable by this data decomposition mechanism. All calculations were performed with MATLAB 2011a (The Mathworks, Natick, MA, USA) equipped with the PLS Toolbox v. 6.2.1 (Eigenvector Research Inc., Wenatchee, WA, USA). In this analysis, the last 50 ns of MD simulation trajectory data of different potential energy were used. Before applying PCA, autoscale function was used to preprocess the data. First 4 PCs explained >80% of the energy variation.

## Supplementary information

Supporting information

## Data Availability

The datasets generated during and/or analysed during the current study are available from the corresponding author on reasonable request.

## References

[1] Lee J, Ha J, Hyun J, Goo M (2005). Gene SNPs and mutations in clinical genetic testing: haplotype-based testing and analysis. Mutat. Res..

[2] Rajasekaran R (2008). G. P. D. C. Computational and Structural Investigation of Deleterious Functional SNPs in Breast Cancer BRCA2 Gene. Chin. J. Biotechnol..

[3] George Priya Doss C (2008). A novel computational and structural analysis of nsSNPs in CFTR gene. Genomic Med..

[4] Chitrala, K. N., Yeguvapalli, S., Screening, C. & Dynamic, M. Computational Screening and Molecular Dynamic Simulation of Breast Cancer Associated Deleterious Non- Synonymous Single Nucleotide Polymorphisms in TP53. *PLoS One* **9** (2014). 10.1371/journal.pone.0104242 PMC4126775 25105660

[5] Jia M (2014). Computational analysis of functional single nucleotide polymorphisms associated with the CYP11B2 gene. PLoS One.

[6] Ramensky V, Bork P, Sunyaef S (2002). Human non-synonymous SNPs: server and survey. Nucleic Acids Res..

[7] Radivojac P (2011). Identification, Analysis and Prediction of Protein Ubiquitination Sites. Proteins.

[8] Doniger Scott W., Kim Hyun Seok, Swain Devjanee, Corcuera Daniella, Williams Morgan, Yang Shiaw-Pyng, Fay Justin C. (2008). A Catalog of Neutral and Deleterious Polymorphism in Yeast. PLoS Genetics.

[9] Barroso I (1999). Dominant negative mutations in human PPARgamma associated with severe insulin resistance, diabetes mellitus and hypertension. Nature.

[10] Chasman D, Adams RM (2001). Predicting the functional consequences of non-synonymous single nucleotide polymorphisms: structure-based assessment of amino acid variation. J. Mol. Biol..

[11] Ng PC, Henikoff S (2006). Predicting the Effects of Amino Acid Substitutions on Protein Function. Annu. Rev. Genomics Hum. Genet..

[12] Wang, Z. *et al*. A novel mutation in STK11 gene is associated with Peutz-Jeghers Syndrome in Chinese patients. *BMC Med. Genet*. **12**, 161 (2011). 10.1186/1471-2350-12-161 PMC3297525 22168747

[13] Tiainen M, Vaahtomeri K, Ylikorkala A, Mäkelä TP (2002). Growth arrest by the LKB1 tumor suppressor: induction of p21(WAF1/CIP1). Hum. Mol. Genet..

[14] Ylikorkala A (2001). Linked references are available on JSTOR for this article: Deregulation of VEGF in Lkbl-Deficient Mice. Science (80-.)..

[15] Marignani PA, Kanai F, Carpenter CL (2001). LKB1 Associates with Brg1 and Is Necessary for Brg1-induced Growth Arrest. J. Biol. Chem..

[16] Boudeau J (2004). Analysis of the LKB1-STRAD-MO25 complex. J. Cell Sci..

[17] Kopacova M, Tacheci I, Rejchrt S, Bures J (2009). Peutz-Jeghers syndrome: Diagnostic and therapeutic approach. World J. Gastroenterol..

[18] Fan RY, Sheng JQ (2015). A case of Peutz-Jeghers syndrome associated with high-grade intramucosal neoplasia. Int. J. Clin. Exp. Pathol..

[19] Lier MGFV, Wagner A, Kuipers EJ, Steyerberg EW (2010). High Cancer Risk in Peutz – Jeghers Syndrome: A Systematic Review and Surveillance Recommendations. Am. J. Gastroenterol..

[20] Weng M, Ni Y, Su Y, Wong J, Wei S (2010). Clinical and Genetic Analysis of Peutz – Jeghers Syndrome Patients in Taiwan. J. Formos. Med. Assoc..

[21] Orellana P, López-Köstner F, Heine C, Suazo C, Pinto E, Church J, Carvallo P, Alvarez K (2013). Large deletions and splicing-site mutations in the STK11 gene in Peutz-Jeghers Chilean families. Clinical Genetics.

[22] Volikos E (2006). LKB1 exonic and whole gene deletions are a common cause of Peutz-Jeghers syndrome. J. Med. Genet..

[23] Aretz S (2005). High Proportion of Large Genomic STK11 Deletions in Peutz-Jeghers Syndrome. Hum. Mutat..

[24] Lino Cardenas CL (2011). Genetic polymorphism of CYP4A11 and CYP4A22 genes and in silico insights from comparative 3D modelling in a French population. Gene.

[25] Rabbani B, Mahdieh N, Haghi Ashtiani MT, Setoodeh A, Rabbani A (2012). In silico structural, functional and pathogenicity evaluation of a novel mutation: An overview of HSD3B2 gene mutations. Gene.

[26] Hussain MRM (2012). In silico analysis of Single Nucleotide Polymorphisms (SNPs) in human BRAF gene. Gene.

[27] Baynes C (2007). Common variants in the ATM, BRCA1, BRCA2, CHEK2 and TP53 cancer susceptibility genes are unlikely to increase breast cancer risk. Breast Cancer Res..

[28] Doss CGP, Sethumadhavan R (2009). Investigation on the role of nsSNPs in HNPCC genes–a bioinformatics approach. J. Biomed. Sci..

[29] de Alencar S. A., Lopes Julio C. D. (2010). A ComprehensiveIn SilicoAnalysis of the Functional and Structural Impact of SNPs in theIGF1RGene. Journal of Biomedicine and Biotechnology.

[30] Kumar A, Rajendran V, Sethumadhavan R, Purohit R (2013). Evidence of Colorectal Cancer-Associated Mutation in MCAK: A Computational Report. Cell Biochem. Biophys..

[31] Scott RJ (2002). Mutation analysis of the STK11/LKB1 gene and clinical characteristics of an Australian series of Peutz-Jeghers syndrome patients. Clin. Genet..

[32] Ashkenazy H, Erez E, Martz E, Pupko T, Ben-tal N (2010). ConSurf 2010: calculating evolutionary conservation in sequence and structure of proteins and nucleic acids. Nucleic Acids Res..

[33] Zeqiraj E, Filippi BM, Deak M, Alessi DR, Aalten DMF (2009). Van. Structure of the LKB1-STRAD-MO25 Complex Reveals an Allosteric Mechanism of Kinase Activation. Science (80-.)..

[34] Dilmeç F, Varışlı L, Özgönül A, Cen O (2007). Analysis of Stk11 / Lkb1 Gene Using Bioinformatics Tools. Eur J Gen Med.

[35] Deventer SJH (2000). van. Cytokine and cytokine receptor polymorphisms in infectious disease. Intensive Care Med..

[36] Pesole G (1999). Internet resources for the functional analysis of 5 Ј and 3 Ј untranslated regions of eukaryotic mRNAs. Resour. Internet.

[37] Mehenni H (1998). Loss of LKB1 Kinase Activity in Peutz-Jeghers Syndrome, and Evidence for Allelic and Locus Heterogeneity. Am. J. Hum. Genet..

[38] Guex N, Peitsch MC (1997). SWISS-MODEL and the Swiss-Pdb Viewer: An environment for comparative protein modeling. Electrophoresis.

[39] Sci JC (2012). The Role of Arg157Ser in Improving the Compactness and Stability of ARM Lipase. J. Comput. Sci. Syst. Biol..

[40] Gilis D, Rooman M (1996). Stability Changes upon Mutation of Solvent- accessible Residues in Proteins Evaluated by Database-derived Potentials. J. Mol. Biol..

[41] Greene LH (2001). Role of conserved residues in structure and stability: Tryptophans of human serum retinol-binding protein, a model for the lipocalin superfamily. Protein Sci..

[42] Williamson K (2013). Catalytic and Functional Roles of Conserved Amino Acids in the SET Domain of the S. cerevisiae Lysine Methyltransferase Set1. PLoS One.

[43] Xu Y, Wang H, Nussinov R, Program BS (2013). Protein Charge and Mass Contribute to the Spatio-temporal Dynamics of Protein-Protein Interactions in a Minimal Proteome. Proteomics.

[44] Peleg O, Choi J, Shakhnovich EI (2014). Evolution of Specificity in Protein-Protein Interactions. Biophys. J..

[45] Rodriguez-Casado A (2012). In silico investigation of functional nsSNPs – an approach to rational drug design. Res. Reports Med. Chem..

[46] Rungsung, I. & Ramaswamy, A. Insights into the structural dynamics of Liver kinase B1 (LKB1) by the binding of STe20 Related Adapter α (STRAD alpha) and Mouse protein 25alpha (MO25alpha) co-activators. *J*. *Biomol*. *Struct*. *Dyn*. **1102** (2016). 10.1080/07391102.2016.1173593 27160967

[47] Zheng J (1993). Crystal Structure of the Catalytic Subunit of CAMP-Dependent Protein Kinase Complexed with MgATP and Peptide Inhibitor. Biochemistry.

[48] Pace CN (2014). Contribution of hydrogen bonds to protein stability. Protein Sci..

[49] Pace CN (2012). Contribution of Hydrophobic Interactions to Protein Stability. J. Mol. Biol..

[50] Rungsung I, Ramaswamy A (2017). Insights into the structural dynamics of Liver kinase B1 (LKB1) by the binding of STe20 Related Adapterα (STRADα) and Mouse protein 25α (MO25α) co-activators. J. Biomol. Struct. Dyn..

[51] Hamosh A, Scott AF, Amberger JS, Bocchini CA, McKusick VA (2005). Online Mendelian Inheritance in Man (OMIM), a knowledgebase of human genes and genetic disorders. Nucleic Acids Res..

[52] Sherry S (2001). T. dbSNP: the NCBI database of genetic variation. Nucleic Acids Res..

[53] Apweiler R (2009). The universal protein resource (UniProt) in 2010. Nucleic Acids Res..

[54] Ng PC, Henikoff S (2003). SIFT: predicting amino acid changes that affect protein function. Nucleic Acids Res..

[55] Adzhubei IA (2010). A method and server for predicting damaging missense mutations a. Nat. Methods.

[56] Choi Y, Sims GE, Murphy S, Miller JR, Chan AP (2012). Predicting the Functional Effect of Amino Acid Substitutions and Indels. PLoS One.

[57] Ferrer-costa C (2005). Structural bioinformatics PMUT: a web-based tool for the annotation of pathological mutations on proteins. Bioinformatics.

[58] Bromberg Y, Yachdav G, Rost B (2008). SNAP predicts effect of mutations on protein function. Bioinformatics.

[59] Niroula A, Urolagin S, Vihinen M (2015). PON-P2: Prediction Method for Fast and Reliable Identification of Harmful Variants. PLoS One.

[60] Reva Boris, Antipin Yevgeniy, Sander Chris (2011). Predicting the functional impact of protein mutations: application to cancer genomics. Nucleic Acids Research.

[61] Hunter S (2009). InterPro: The integrative protein signature database. Nucleic Acids Res..

[62] Mayrose I, Graur D, Ben-Tal N, Pupko T (2004). Comparison of site-specific rate-inference methods for protein sequences: Empirical Bayesian methods are superior. Mol. Biol. Evol..

[63] Pupko T, Bell RE, Mayrose I, Glaser F, Ben-Tal N (2002). Rate4Site: an algorithmic tool for the identification of functional regions in proteins by surface mapping of evolutionary determinants within their homologues. Bioinformatics.

[64] Tamura K (2011). MEGA5: Molecular Evolutionary Genetics Analysis Using Maximum Likelihood, Evolutionary Distance, and Maximum Parsimony. Methods. Mol. Evol. Genet. Anal..

[65] Felsenstein J (2012). Confidence Limits On Phylogenies: An Approach Using The Bootstrap Joseph. Evolution (N. Y)..

[66] Pesole G (2001). Structural and functional features of eukaryotic mRNA untranslated regions. Gene.

[67] Grillo G (2010). UTRdb and UTRsite (RELEASE 2010): a collection of sequences and regulatory motifs of the untranslated regions of eukaryotic mRNAs. Nucleic Acids Res..

[68] Doss CGP, Chakraborty C (2013). In silico discrimination of nsSNPs in hTERT gene by means of local DNA sequence context and regularity. J. Mol. Model..

[69] Dassault Systèmes BIOVIA. Discovery Studio Modeling Environment. Release 4.1, San Diego: Dassault Systèmes (2015).

[70] Venselaar H, Ah T, Kuipers RKP, Hekkelman ML, Vriend G (2010). Protein structure analysis of mutations causing inheritable diseases. An e-Science approach with life scientist friendly interfaces. BMC Bioinformatics.

[71] Grate L. R., Bhattacharyya C., Jordan M. I., Mian I. S. (2002). Simultaneous Relevant Feature Identification and Classification in High-Dimensional Spaces. Lecture Notes in Computer Science.

[72] Schneidman-Duhovny D, Inbar Y, Nussinov R, Wolfson HJ (2005). PatchDock and SymmDock: servers for rigid and symmetric docking. Nucleic Acids Res..

[73] MJ, F. Electronic Supplementary Material (ESI) for Chemical Science. *Gaussian 09*, *Revis*. *E*.*01*. *Gaussian*, *Inc*., *Wallingford CT*, *USA* 1–20 (2009).

[74] Trott O, Olson AJ (2010). AutoDock Vina: improving the speed and accuracy of docking with a new scoring function, efficient optimization, and multithreading. J. Comput. Chem..

[75] Patel, H., Grüning, B. A., Günther, S. & Merfort, I. PyWATER: A PyMOL plugin to find conserved water molecules in proteins by clustering. *Struct*. *Bioinforma*. 2–4 (2014). 10.1093/bioinformatics/btu424 24990608

[76] Krieger, E., Dunbrack, R., Hooft, R. & Krieger, B. *Assignment of Protonation States in Proteins and Ligands: Combining pKa Prediction with Hydrogen Bonding Network Optimization*. **819** (2012). 10.1007/978-1-61779-465-0_25 22183550

[77] Dickson CJ (2014). Lipid14: The amber lipid force field. J. Chem. Theory Comput..

[78] Krieger E, Nielsen JE, Spronk CAEM, Vriend G (2006). Fast empirical p K a prediction by Ewald summation. J. Mol. Graph. Model..

[79] Darden T, York D, Pedersen L (1993). Particle mesh Ewald: An *N* ⋅log(*N*) method for Ewald sums in large systems. J. Chem. Phys..

[80] Wold S, Esbensen K, Geladi P (1987). Principal component analysis. Chemom. Intell. Lab. Syst..

[81] Martens, H. & Naes, T. *Multivariate Calibration*. (John Wiley & Sons, Ltd, 1992).

