# Supplementary material for: Prediction of Deleterious Non-synonymous SNPs of Human STK11 Gene by Combining Algorithms, Molecular Docking, and Molecular Dynamics Simulation
Source: Sci Rep. 2019 Nov 11;9:16426. doi: 10.1038/s41598-019-52308-0 (PMC6848484; doi:10.1038/s41598-019-52308-0)
Supplement: Supplementary file 1 — Supporting information [file 41598_2019_52308_MOESM1_ESM.docx]

Prediction of Deleterious Non-synonymous SNPs of Human STK11 Gene by Combining Algorithms, Molecular Docking, and Molecular Dynamics Simulation

**Md. Jahirul Islam^1,2^, Akib Mahmud Khan^1^, Md. Rimon Parves^2^, Nayeem Hossain^1^ and Mohammad A. Halim^1*^**

^1^Division of Computer-Aided Drug Design, The Red-Green Research Centre, BICCB, 218 Elephant Road, Dhaka 1205, Bangladesh

^2^Department of Biochemistry and Biotechnology, University of Science and Technology Chittagong (USTC), Foy’s Lake, Khulshi– 4202, Chittagong, Bangladesh

*Correspondence: mahalim@grc-bd.org

**Table S1**: List of SNPs predicted by SIFT, Polyphen2, I-Mutant and PROVEAN

| **dbSNP** | **Mutation** | **SFIT Score** | **Prediction** | **Polyphen-2 Score** | **Prediction** | **I-Mutant 3.0** | **Score** | **PROVEAN** | **Score** |
| --- | --- | --- | --- | --- | --- | --- | --- | --- | --- |
| **rs730881975** | **W239C** | **0.0** | **Damaging** | **1.0** | **PD** | **Large decrease** | **-1.85** | **Deleterious** | **-12.938** |
| **rs864622488** | **W308C** | **0.0** | **Damaging** | **1.00** | **PD** | **Large decrease** | **-1.24** | **Deleterious** | **-12.462** |
| **rs745622138** | **W239R** | **0.0** | **Damaging** | **1.00** | **PD** | **Large decrease** | **-1.39** | **Deleterious** | **-13.933** |
| rs730881972 | G163R | 0.0 | Damaging | 1.0 | PD | Large decrease | -0.22 | Deleterious | -7.989 |
| rs878853992 | G242W | 0.0 | Damaging | 1.00 | PD | Large decrease | -0.43 | Deleterious | -7.962 |
| rs730881977 | G251D | 0.0 | Damaging | 0.999 | PD | Large decrease | -1.16 | Deleterious | -6.057 |
| rs137853080 | Y49D | 0.0 | Damaging | 1.00 | PD | Large decrease | -0.72 | Deleterious | -9.625 |
| rs137853081 | G135A | 0.0 | Damaging | 0.884 | PD | Large Increase | -0.34 | Deleterious | -3.480 |
| rs730881978 | R297G | 0.0 | Damaging | 1.0 | PD | Large decrease | -1.40 | Deleterious | -6.429 |
| rs367807476 | P324L | 0.05 | Damaging | 0.146 | Benign | Large decrease | -0.26 | Deleterious | -3.459 |
| rs730881979 | D176N | 0.0 | Damaging | 1.0 | PD | Large decrease | -0.89 | Deleterious | -5.000 |
| rs730881980 | H202Y | 0.01 | Damaging | 0.971 | PD | Large Increase | 0.24 | Neutral | -2.457 |
| rs730881984 | R297S | 0.0 | Damaging | 1.00 | PD | Large decrease | -1.18 | Deleterious | -5.505 |
| rs730881989 | E92G | 0.01 | Damaging | 0.896 | PD | Large decrease | -0.83 | Deleterious | -6.369 |
| rs137853078 | G163D | 0.0 | Damaging | 1.0 | PD | Large decrease | -0.78 | Deleterious | -6.990 |
| rs730881992 | R425H | 0.03 | Damaging | 0.999 | PD | Large decrease | -0.92 | Neutral | -1.836 |
| rs137853081 | G135R | 0.00 | Damaging | 1.00 | PD | Large Increase | -0.13 | Deleterious | -5.278 |
| rs748464757 | R147C | 0.0 | Damaging | 0.999 | PD | Large decrease | -1.02 | Deleterious | -5.695 |
| rs730881973 | L182M | 0.00 | Damaging | 0.993 | PD | Large decrease | -0.94 | Neutral | -1.933 |
| rs730881974 | L182P | 0.00 | Damaging | 1.0 | PD | Large decrease | -1.23 | Deleterious | -6.867 |
| rs750708224 | E14G | 0.01 | Damaging | 0.346 | Begin | Large decrease | -1.05 | Neutral | -1.228 |
| rs185087320 | R211W | 0.01 | Damaging | 0.998 | PD | Large decrease | 0.02 | Neutral | -2.095 |
| rs752699287 | R426P | 0.01 | Damaging | 0.513 | PD | Large decrease | -0.37 | Neutral | -1.358 |
| rs200078204 | S404F | 0.04 | Damaging | 0.055 | Begin | Large Increase | 0.53 | Neutral | -1.747 |
| rs368993118 | G61D | 0.00 | Damaging | 1.00 | PD | Large decrease | -0.84 | Deleterious | -6.506 |
| rs375622587 | R106L | 0.04 | Damaging | 0.801 | PD | Large decrease | -0.61 | Deleterious | -3.894 |
| rs376718324 | R405W | 0.01 | Damaging | 0.566 | PD | Large decrease | -0.11 | Neutral | -1.832 |
| rs398123405 | E293K | 0.04 | Damaging | 0.281 | Benign | Large decrease | -0.72 | Neutral | -2.410 |
| rs540627331 | T185I | 0.01 | Damaging | 1.00 | PD | Large decrease | -0.30 | Deleterious | -5.514 |
| rs545015076 | N119D | 0.04 | Damaging | 0.001 | Benign | Large decrease | -0.25 | Deleterious | -2.837 |
| rs549474196 | P324R | 0.03 | Damaging | 0.971 | PD | Large decrease | -0.68 | Deleterious | -3.830 |
| rs567896256 | P339L | 0.03 | Damaging | 0.993 | PD | Large decrease | -0.63 | Deleterious | -7.745 |
| rs587780012 | R301W | 0.01 | Damaging | 0.028 | Benign | Large decrease | -0.37 | Deleterious | -3.149 |
| rs587780717 | R147H | 0.03 | Damaging | 0.107 | Benign | Large decrease | -1.51 | Deleterious | -3.506 |
| rs587781179 | Q432H | 0.02 | Damaging | 0.938 | PD | Large decrease | -0.63 | Neutral | -1.189 |
| rs587781303 | Y126H | 0.00 | Damaging | 1.00 | PD | Large decrease | -1.20 | Deleterious | -4.960 |
| rs587781537 | S428W | 0.00 | Damaging | 1.00 | PD | Large Increase | 0.16 | Deleterious | -2.772 |
| rs587781638 | G233D | 0.00 | Damaging | 1.00 | PD | Large decrease | -1.28 | Deleterious | -6.967 |
| rs587781638 | G233S | 0.00 | Damaging | 1.00 | PD | Large decrease | -1.51 | Deleterious | -5.971 |
| rs587782115 | R106W | 0.01 | Damaging | 1.00 | PD | Large decrease | -0.34 | Deleterious | -4.457 |
| rs587782146 | P217L | 0.00 | Damaging | 1.00 | PD | Large decrease | -0.24 | Deleterious | -9.933 |
| rs587782546 | P221L | 0.03 | Damaging | 1.00 | PD | Large decrease | -0.27 | Deleterious | -9.357 |
| rs587782783 | R104W | 0.03 | Damaging | 1.00 | PD | Large decrease | -0.31 | Deleterious | -4.450 |
| rs727504171 | I303S | 0.00 | Damaging | 1.00 | PD | Large decrease | -1.80 | Deleterious | -5.273 |
| rs730881970 | Q137K | 0.00 | Damaging | 0.995 | PD | Large Increase | -0.18 | Deleterious | -3.900 |
| rs730881976 | S240W | 0.00 | Damaging | 1.00 | PD | Large Increase | -0.05 | Deleterious | -6.467 |
| rs763353991 | G155W | 0.02 | Damaging | 1.00 | PD | Large decrease | -0.23 | Deleterious | -3.956 |
| rs771765869 | D30E | 0.01 | Damaging | 0.640 | PD | Large Increase | -0.13 | Deleterious | -2.814 |
| rs773422811 | A76V | 0.0 | Damaging | 1.00 | PD | Large Increase | 0.03 | Deleterious | -3.849 |
| rs786201090 | R304W | 0.01 | Damaging | 1.00 | PD | Large decrease | -0.35 | Deleterious | -5.585 |
| rs786202134 | L195M | 0.0 | Damaging | 0.999 | PD | Large decrease | -1.18 | Neutral | -0.800 |
| rs786202466 | P179L | 0.03 | Damaging | 1.00 | PD | Large decrease | -0.60 | Deleterious | -9.667 |
| rs786203250 | R39L | 0.01 | Damaging | 0.921 | PD | Large decrease | -0.52 | Deleterious | -3.466 |
| rs864622638 | D327G | 0.03 | Damaging | 0.381 | Benign | Large Increase | -0.88 | Neutral | 0.244 |
| rs876658594 | R331W | 0.05 | Damaging | 0.003 | Benign | Large decrease | -0.45 | Neutral | -2.344 |
| rs876658779 | R39C | 0.0 | Damaging | 1.00 | PD | Large decrease | -1.10 | Deleterious | -4.194 |
| rs376280361 | R304Q | 0.05 | Damaging | 0.960 | PD | Large decrease | -0.89 | Neutral | -1.907 |
| rs750366043 | R310W | 0.02 | Damaging | 0.999 | PD | Large decrease | -0.12 | Deleterious | -3.239 |
| rs758416485 | P314L | 0.01 | Damaging | 0.002 | Benign | Large Increase | -0.05 | Deleterious | -6.504 |
| rs775595174 | E120K | 0.04 | Damaging | 0.146 | Benign | Large decrease | -0.61 | Deleterious | -3.116 |
| rs778868074 | C134W | 0.01 | Damaging | 1.00 | PD | Large decrease | -0.10 | Neutral | -2.333 |
| rs866454760 | P294Q | 0.0 | Damaging | 0.997 | PD | Large decrease | -1.24 | Deleterious | -5.533 |
| rs867060159 | E138G | 0.02 | Damaging | 0.072 | Benign | Large decrease | -1.18 | Deleterious | -4.767 |

*PD= Probably Damaging

**Table S2**: List of SNPs predicted by P-Mut, SNAP2, PON-P, and Mutation Assessor algorithms.

| **dbSNP** | **Mutation** | **P-Mut**  **Prediction** | **Score** | **SNAP2**  **Prediction** | **Score** | **PON-P**  **Prediction** | **Score** | **Mutation Assessor** | **Score** |
| --- | --- | --- | --- | --- | --- | --- | --- | --- | --- |
| **rs730881975** | **W239C** | **Disease** | **0.81** | **Effect** | **86** | **Pathogenic** | **0.983** | **High** | **3.885** |
| **rs864622488** | **W308C** | **Disease** | **0.86** | **Effect** | **48** | **Pathogenic** | **0.966** | **Medium** | **2.785** |
| **rs745622138** | **W239R** | **Disease** | **0.81** | **Effect** | **97** | **Unknown** | **0.854** | **High** | **4.435** |
| rs730881972 | G163R | Disease | 0.81 | Effect | 88 | Pathogenic | 0.978 | High | 3.85 |
| rs878853992 | G242W | Disease | 0.81 | Effect | 90 | Pathogenic | 0.935 | High | 4.69 |
| rs730881977 | G251D | Disease | 0.81 | Effect | 84 | Pathogenic | 0.885 | Medium | 2.825 |
| rs137853080 | Y49D | Disease | 0.86 | Effect | 72 | Pathogenic | 0.955 | Medium | 2.98 |
| rs137853081 | G135A | Disease | 0.83 | Effect | 51 | Unknown | 0.607 | Medium | 3.17 |
| rs730881978 | R297G | Disease | 0.83 | Effect | 93 | Pathogenic | 0.942 | High | 4.065 |
| rs367807476 | P324L | Neutral | 0.25 | Neutral | -32 | Unknown | 0.669 | Medium | 1.955 |
| rs730881979 | D176N | Disease | 0.81 | Effect | 89 | Pathogenic | 0.966 | Medium | 2.8 |
| rs730881980 | H202Y | Neutral | 0.28 | Neutral | -8 | Pathogenic | 0.774 | Neutral | 0.79 |
| rs730881984 | R297S | Disease | 0.83 | Effect | 88 | Pathogenic | 0.889 | High | 4.065 |
| rs730881989 | E92G | Disease | 0.56 | Effect | 58 | Pathogenic | 0.866 | Low | 1.545 |
| rs137853078 | G163D | Disease | 0.83 | Effect | 81 | Pathogenic | 0.976 | High | 3.505 |
| rs730881992 | R425H | Disease | 0.68 | Effect | 48 | Unknown | 0.720 | Medium | 1.955 |
| rs137853081 | G135R | Disease | 0.76 | Effect | 68 | Pathogenic | 0.808 | Medium | 3.04 |
| rs748464757 | R147C | Disease | 0.64 | Effect | 52 | Pathogenic | 0.858 | Medium | 2.2 |
| rs730881973 | L182M | Neutral | 0.49 | Neutral | -31 | Unknown | 0.828 | Low | 1.155 |
| rs730881974 | L182P | Disease | 0.84 | Effect | 81 | unknown | 0.856 | Medium | 2.475 |
| rs750708224 | E14G | Neutral | 0.28 | Neutral | -24 | Unknown | 0.464 | Low | 1.87 |
| rs185087320 | R211W | Neutral | 0.25 | Effect | 29 | Pathogenic | 0.805 | Medium | 1.955 |
| rs752699287 | R426P | Disease | 0.50 | Effect | 69 | Pathogenic | 0.799 | Low | 1.78 |
| rs200078204 | S404F | Neutral | 0.50 | Effect | 7 | Unknown | 0.443 | Low | 1.5 |
| rs368993118 | G61D | Disease | 0.78 | Effect | 89 | Pathogenic | 0.977 | Medium | 3.015 |
| rs375622587 | R106L | Disease | 0.73 | Effect | 40 | Unknown | 0.603 | Low | 1.91 |
| rs376718324 | R405W | Neutral | 0.23 | Effect | 31 | Unknown | 0.510 | Low | 0.895 |
| rs398123405 | E293K | Neutral | 0.23 | Effect | 39 | Unknown | 0.516 | Low | 1.02 |
| rs540627331 | T185I | Disease | 0.75 | Effect | 64 | Pathogenic | 0.826 | Medium | 2.06 |
| rs545015076 | N119D | Neutral | 0.10 | Neutral | -63 | Unknown | 0.752 | Neutral | -0.16 |
| rs549474196 | P324R | Neutral | 0.36 | Neutral | -3 | Pathogenic | 0.777 | Medium | 1.955 |
| rs567896256 | P339L | Neutral | 0.24 | Neutral | 0 | Pathogenic | 0.963 | Medium | 2.005 |
| rs587780012 | R301W | Neutral | 0.49 | Effect | 54 | Unknown | 0.765 | Low | 1.41 |
| rs587780717 | R147H | Neutral | 0.35 | Effect | 58 | Unknown | 0.526 | Low | 1.57 |
| rs587781179 | Q432H | Neutral | 0.18 | Neutral | -70 | Unknown | 0.578 | Low | 1.1 |
| rs587781303 | Y126H | Neutral | 0.38 | Effect | 79 | Pathogenic | 0.773 | Low | 1.6 |
| rs587781537 | S428W | Disease | 0.63 | Effect | 31 | Unknown | 0.590 | Medium | 1.955 |
| rs587781638 | G233D | Disease | 0.84 | Effect | 66 | Pathogenic | 0.983 | Low | 1.23 |
| rs587781638 | G233S | Disease | 0.84 | Effect | 24 | Pathogenic | 0.954 | Neutral | 0.585 |
| rs587782115 | R106W | Disease | 0.73 | Effect | 74 | Unknown | 0.747 | Medium | 2.805 |
| rs587782146 | P217L | Neutral | 0.36 | Effect | 67 | Pathogenic | 0.926 | Neutral | 0.34 |
| rs587782546 | P221L | Disease | 0.75 | Effect | 33 | Pathogenic | 0.926 | Medium | 1.98 |
| rs587782783 | R104W | Disease | 0.81 | Effect | 70 | Pathogenic | 0.881 | Medium | 2.14 |
| rs727504171 | I303S | Disease | 0.86 | Effect | 56 | Pathogenic | 0.938 | Medium | 1.98 |
| rs730881970 | Q137K | Neutral | 0.45 | Effect | 71 | Pathogenic | 0.767 | Neutral | 0.65 |
| rs730881976 | S240W | Disease | 0.84 | Effect | 87 | Pathogenic | 0.829 | High | 4.525 |
| rs763353991 | G155W | Neutral | 0.34 | Effect | 36 | Unknown | 0.664 | Low | 1.31 |
| rs771765869 | D30E | Neutral | 0.28 | Neutral | -15 | Unknown | 0.721 | Medium | 2.36 |
| rs773422811 | A76V | Disease | 0.81 | Effect | 34 | Unknown | 0.794 | Medium | 2.34 |
| rs786201090 | R304W | Neutral | 0.21 | Effect | 40 | Pathogenic | 0.888 | Low | 1.54 |
| rs786202134 | L195M | Neutral | 0.48 | Effect | 42 | Pathogenic | 0.756 | Low | 1.14 |
| rs786202466 | P179L | Neutral | 0.33 | Neutral | -60 | Pathogenic | 0.944 | Medium | 1.945 |
| rs786203250 | R39L | Neutral | 0.43 | Effect | 32 | Pathogenic | 0.792 | Medium | 2.24 |
| rs864622638 | D327G | Disease | 0.50 | Effect | -27 | Unknown | 0.365 | Low | 1.7 |
| rs876658594 | R331W | Neutral | 0.33 | Effect | 32 | Pathogenic | 0.846 | Medium | 1.955 |
| rs876658779 | R39C | Disease | 0.60 | Effect | 22 | Pathogenic | 0.862 | Medium | 2.24 |
| rs376280361 | R304Q | Neutral | 0.21 | Neutral | -18 | Unknown | 0.685 | Neutral | 0.52 |
| rs750366043 | R310W | Disease | 0.66 | Effect | 71 | Pathogenic | 0.912 | Medium | 2.265 |
| rs758416485 | P314L | Neutral | 0.46 | Neutral | -25 | Unknown | 0.694 | Medium | 1.955 |
| rs775595174 | E120K | Disease | 0.67 | Effect | -46 | Unknown | 0.648 | Low | 1.375 |
| rs778868074 | C134W | Disease | 0.61 | Effect | 61 | Unknown | 0.648 | Low | 1.215 |
| rs866454760 | P294Q | Disease | 0.86 | Effect | 58 | Unknown | 0.635 | Medium | 2.095 |
| rs867060159 | E138G | Disease | 0.84 | Effect | 39 | Pathogenic | 0.795 | Low | 1.465 |


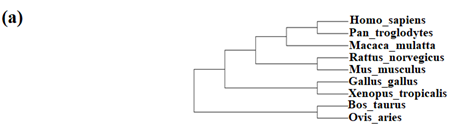


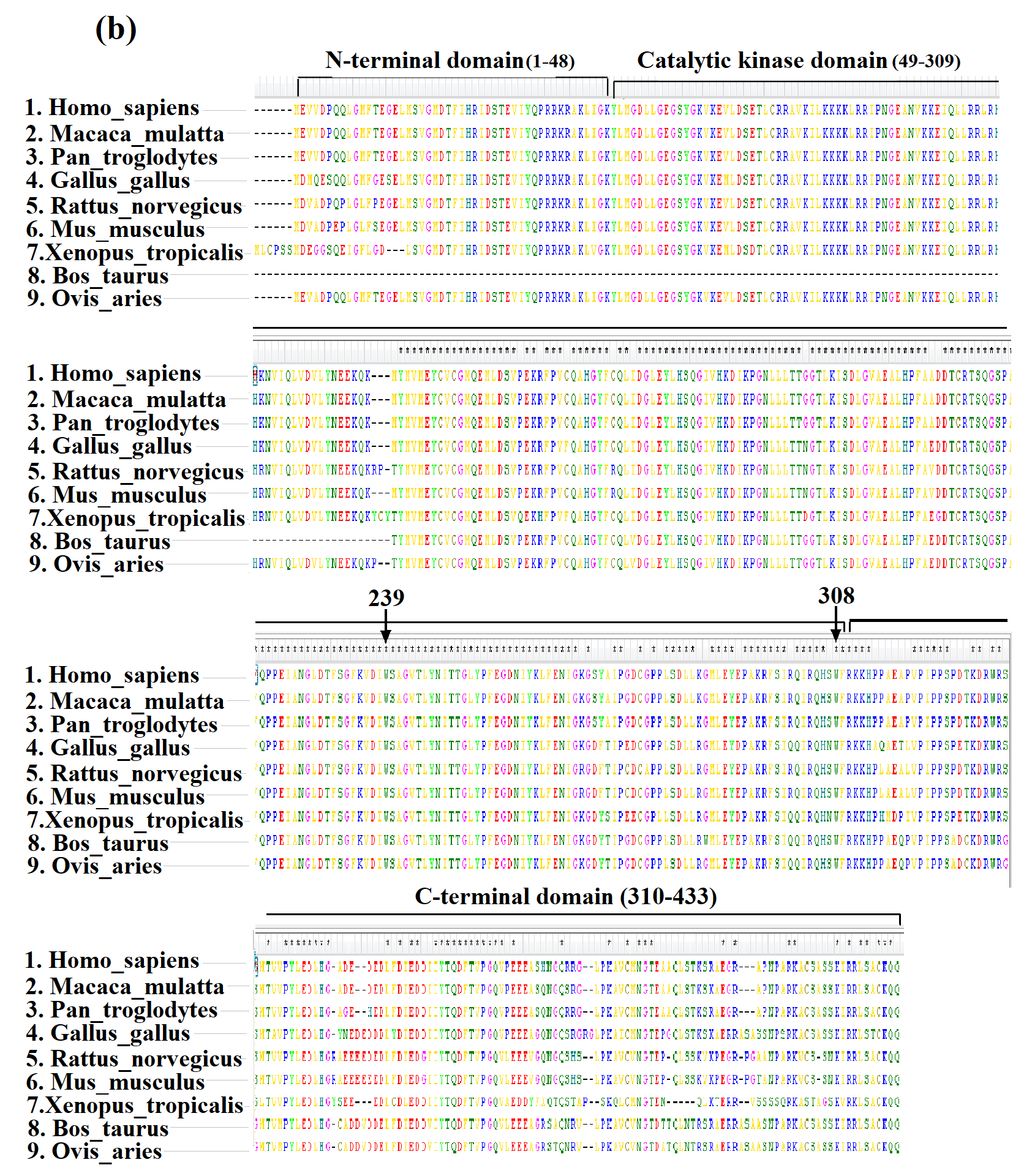


**Figure S1:** (a) Phylogenetic analysis of STK11 protein. (b) Multiple sequence alignments and evolutionary conservation behavior within STK11 protein. W239 and W308 residues are found conserved in the catalytic kinase domain (as shown with “*”).

**Table S3**: Results showing UTR regions in *STK11* transcript from UTRScan server.

| Signal name | UTR region | Total match | Position in transcript |
| --- | --- | --- | --- |
| uorf (upstream open reading frames) | 5 | 2 | 40-237  295-390 |
| ADH_DRE (Alcohol dehydrogenase 3'UTR downregulation control element) | 3 | 1 | 331-338 |


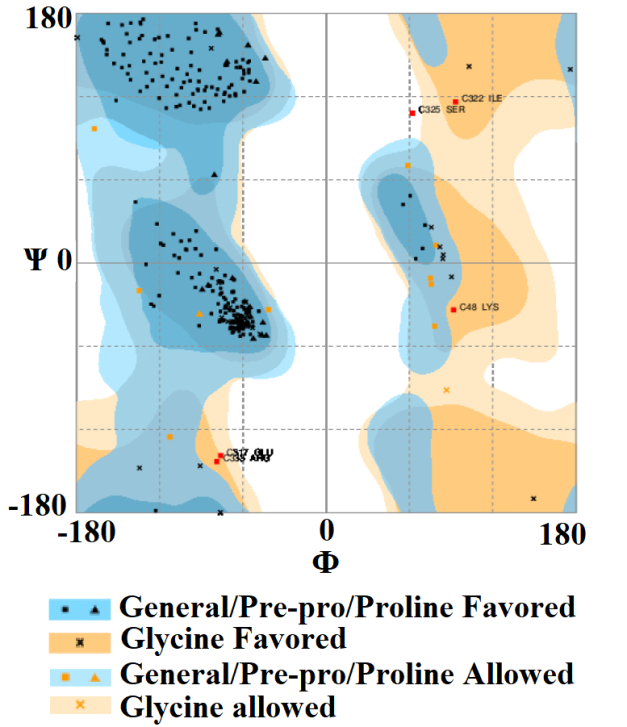


**Figure S2:** Ramachandran plot created by RAMPAGE for STK11.

**Table S4:** Analysis of Ramachandran plot of modelled structures using RAMPAGE server.

| Model | Residues in most favored regions | | Residues in allowed regions | | Residue in outlier region | |
| --- | --- | --- | --- | --- | --- | --- |
|  | No. of residues | % of residues | No. of residues | % of residues | No. of residues | % of residues |
| Wild | 278 | 94.6 | 11 | 3.7 | 5 | 1.7 |
| W239R | 278 | 94.6 | 11 | 3.7 | 5 | 1.7 |
| W308C | 278 | 94.6 | 11 | 3.7 | 5 | 1.7 |


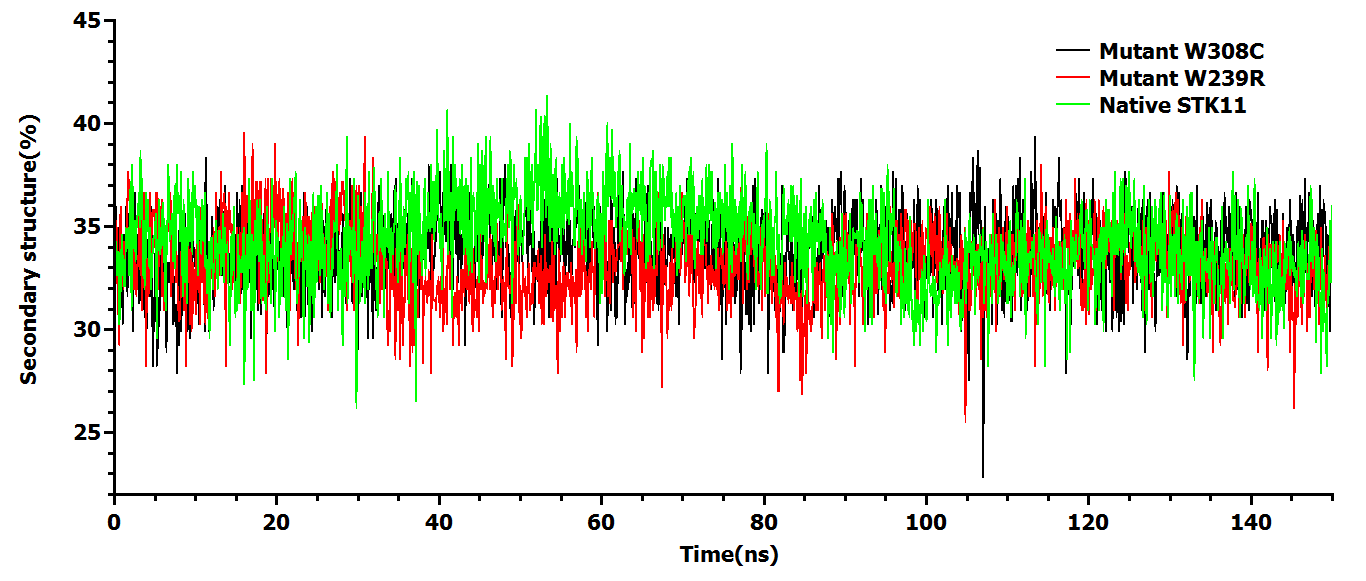


**a)**

**b)**

**c)**

**d)**


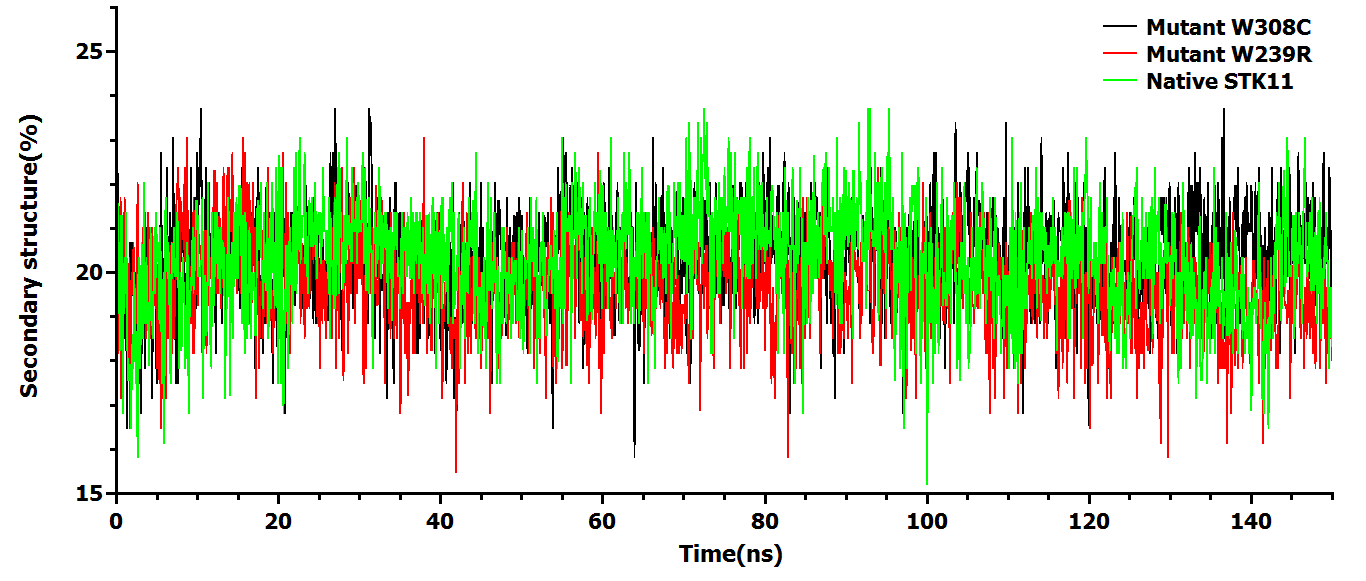


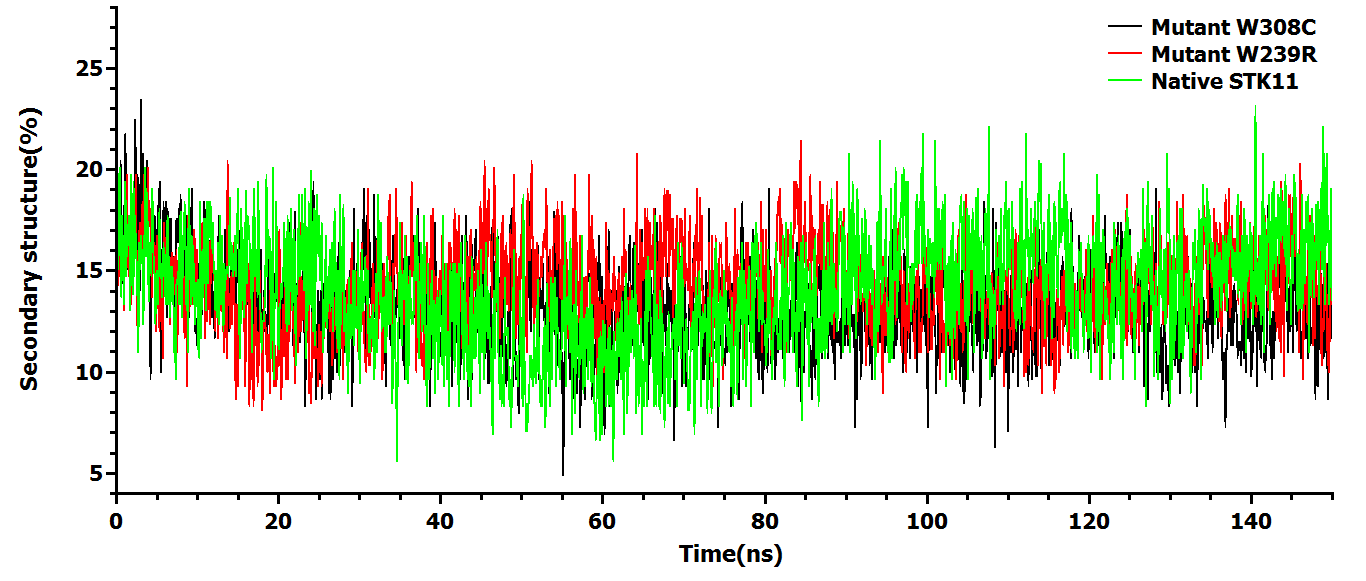


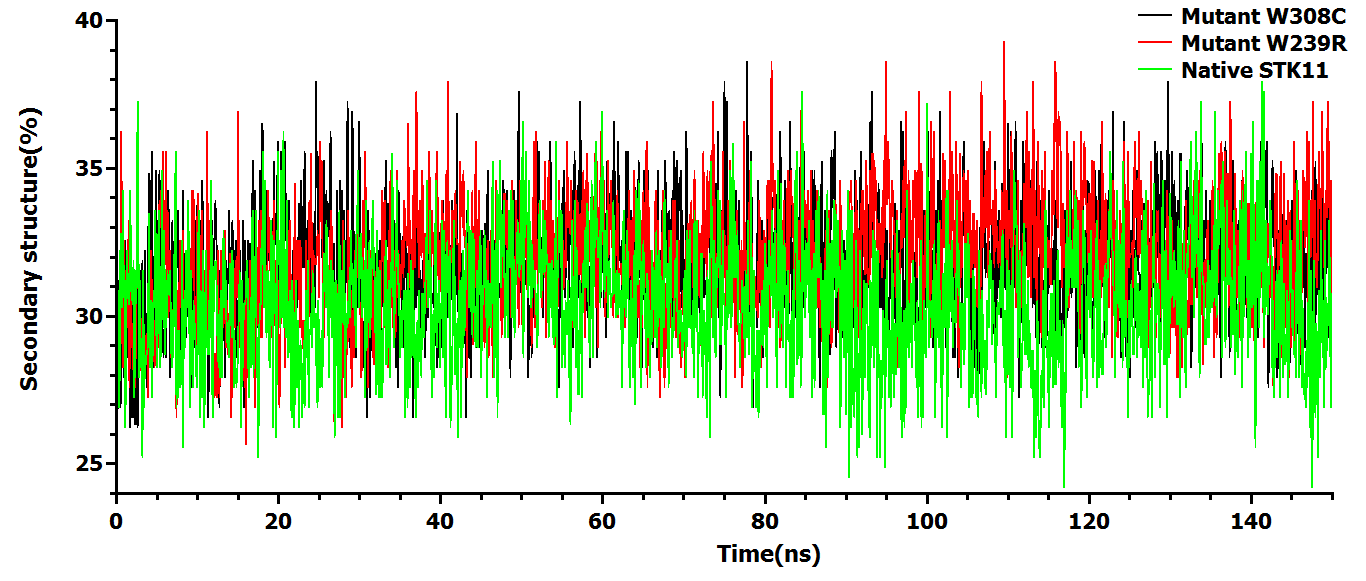


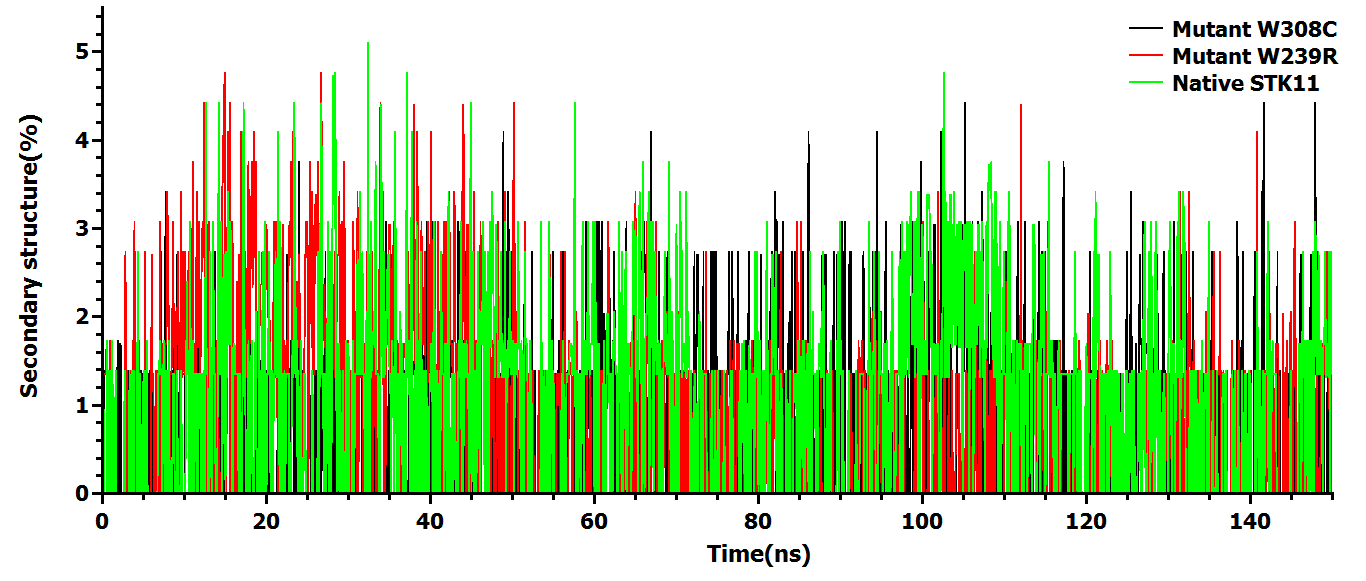


**e)**

**Figure S3:** Secondary structure comparison between native STK11 and mutants. Here, (a) helix, (b) sheet, (c) turn, (d) coil, and

(e) 3_10_ helix.
